# Unveiling cryptic diversity of *Diaporthe* associated with leaf spots of *Fagaceae* in China using an integrative taxonomic approach

**DOI:** 10.3897/imafungus.17.186438

**Published:** 2026-05-15

**Authors:** Xue Zhong, Hermann Voglmayr, Han Xue, Yong Li, Ning Jiang

**Affiliations:** 1 Key Laboratory of Forest Protection of National Forestry and Grassland Administration, Ecology and Nature Conservation Institute, Chinese Academy of Forestry, Beijing 100091, China Key Laboratory of Forest Protection of National Forestry and Grassland Administration, Ecology and Nature Conservation Institute, Chinese Academy of Forestry Beijing China; 2 Department of Botany and Biodiversity Research, University of Vienna, Vienna, Austria Department of Botany and Biodiversity Research, University of Vienna Vienna Austria

**Keywords:** *
Ascomycota
*, cryptic species, foliar pathogens, multi-locus phylogeny, *
Sordariomycetes
*, Southern China, taxonomy

## Abstract

The genus *Diaporthe* comprises a functionally diverse group of fungi, inhabiting plant tissues as pathogens, endophytes, and saprobes. Many species cause significant diseases in economically and ecologically important woody plants. Despite extensive research on the taxonomy and phylogeny of *Diaporthe* over the past two decades, species delineation remains challenging due to the high morphological plasticity among the more than 1,000 described taxa. In practice, molecular phylogenetic divergence has become the primary criterion for species delimitation, as morphological characters often fail to resolve species boundaries. In this study, we employed an integrative taxonomic approach combining multi-locus phylogeny with morphological characterization to investigate *Diaporthe* isolates associated with leaf spot diseases on *Fagaceae*, a dominant tree family in Chinese forests. Our investigation resolved eight distinct lineages, including five novel species described herein: namely *D. castanopsidicola*, *D. changjiangensis*, *D. cyclobalanopsidicola*, *D. liangxii*, and *D. lithocarpicola* from Guangdong and Hainan Provinces, China. These findings significantly contribute to resolving the cryptic diversity of *Diaporthe* and expand our understanding of the fungal communities driving foliar diseases in *Fagaceae*.

## Introduction

The genus *Diaporthe* (syn. *Phomopsis*), a hyper-diverse lineage within the *Diaporthaceae (Diaporthales)*, represents one of the most ecologically successful groups of *Ascomycota* (Voglmayr et al. 2012; Dissanayake et al. 2017; Fan et al. 2020; Udayanga et al. 2021; Jiang et al. 2023, 2024; Zhu et al. 2023). Members of this genus are characterized by typical ordinal features, particularly in the teleomorph, such as immersed ascomata with elongated perithecial necks, and unitunicate, clavate to cylindrical asci containing hyaline, fusoid to cylindrical ascospores (Dissanayake et al. 2017; Yang et al. 2018; Jiang et al. 2021). In the anamorphic state, *Diaporthe* is identified by ostiolate conidiomata and cylindrical phialides producing three distinct types of hyaline, aseptate conidia (alpha, beta, and gamma) (Dissanayake et al. 2017; Jiang et al. 2021; Liu et al. 2024). While these morphological traits occur in several related taxa, *Diaporthe* forms a robust, monophyletic clade clearly distinguishable through molecular phylogenetic analyses (Voglmayr et al. 2017; Fan et al. 2018; Senanayake et al. 2018; Yang et al. 2020; Cai et al. 2024; Jiang et al. 2025a, 2025b).

Ecologically, *Diaporthe* species occupy a complex niche continuum, functioning as endophytes, saprobes, or opportunistic pathogens (Gomes et al. 2013; Huang et al. 2015; Gao et al. 2016; Cao et al. 2022). Many species are notorious pathogens of economically important woody plants, causing devastating diseases such as leaf spots, fruit rots, branch cankers, and dieback globally (Gomzhina and Gannibal 2022; Bai et al. 2023; Wan et al. 2023). Notable examples include *D. alnicola* and *D. amygdali* on *Alnus* in China (Li et al. 2025), and the *D. ambigua* complex associated with kiwifruit rot in Chile (Díaz et al. 2017). Similarly, *D. foeniculina* and allied species have been implicated in severe shoot blights in crops ranging from citrus to blueberry across Europe (Vakalounakis et al. 2019; Hilário et al. 2020).

Given its economic and ecological significance, the taxonomy of *Diaporthe* has been a focal point of mycology since the genus was established (Nitschke 1870). Historically, species identification relied heavily on host association and morphology (Mostert et al. 2001; Santos and Phillips 2009). However, modern investigations have revealed that high morphological plasticity and broad host ranges render traditional identification unreliable (Jiang et al. 2021; Zhu et al. 2024). Consequently, the field now employs integrative taxonomy. Recent studies employing genealogical concordance phylogenetic species recognition (GCPSR) and coalescence-based models, such as the General Mixed Yule-Coalescent (GMYC) and Poisson Tree Processes (PTP), have been instrumental in resolving cryptic species complexes and refining the generic boundaries (Hilário et al. 2020, 2021a, 2021b; Dissanayake et al. 2024; Pereira and Phillips 2024). These advanced approaches resulted in both the description of numerous novel lineages and the necessary synonymization of previously over-described taxa (Ren et al. 2025).

Despite these advances, knowledge of *Diaporthe* diversity remains uneven across host families. *Fagaceae*, comprising dominant tree species (e.g., *Castanopsis*, *Lithocarpus*, *Quercus*) in the subtropical evergreen broad-leaved forests of China, plays a critical role in forest ecosystem structure and carbon cycling. While leaf spot diseases are frequently observed in these forests, the diversity and taxonomy of the associated *Diaporthe* species remain poorly understood. This study aims to bridge this gap by (1) conducting a systematic survey of *Diaporthe* associated with leaf spots on *Fagaceae* in China, and (2) applying an integrative taxonomic framework to rigorously delimit species boundaries and unveil cryptic diversity.

## Materials and methods

### Surveys and isolations

In 2019, surveys of leaf spot diseases affecting *Fagaceae* were conducted in Yangshan County, Qingyuan City, Guangdong Province, and Changjiang Li Autonomous County, Hainan Province, China. Symptomatic leaves were collected from nine host species: *Castanopsis chunii*, *C. hystrix*, *Cyclobalanopsis austrocochinchinensis*, *Cy. blakei*, *Cy. glauca*, *Cy. kerrii*, *Lithocarpus amygdalifolius*, *L. levis*, and *L. naiadarum*. Samples were placed in sealed plastic bags and transported to the laboratory. Fungal isolation was performed following surface sterilization. Leaves were rinsed under running tap water to remove surface debris, immersed in 75% ethanol for 3 min, rinsed with sterile distilled water for 2 min, and dried with sterile absorbent cotton. Leaf segments (approx. 5 × 5 mm) containing the margin between diseased and healthy tissue were excised using a sterile scalpel and plated onto potato dextrose agar (PDA; 200 g potatoes, 20 g dextrose, and 20 g agar per L). Plates were incubated at 25 °C in the dark until mycelial growth was observed. Hyphal tips were transferred to fresh PDA plates to obtain pure cultures. Holotype specimens were deposited in the Herbarium of the Chinese Academy of Forestry (CAF; http://museum.caf.ac.cn/), and ex-type living cultures were deposited in the China Forestry Culture Collection Center (CFCC; https://cfcc.caf.ac.cn/).

### Morphological analyses

Cultural characteristics were assessed on PDA, malt peptone agar (MPA; 30 g malt extract, 5 g mycological peptone, and 15 g agar per L), and synthetic nutrient agar (SNA; containing 0.2 g glucose, 0.2 g sucrose, 1 g KH_2_PO_4_, 1 g KNO_3_, 0.25 g MgSO_4_, 0.5 g KCl, and 14 g agar per L). To induce sporulation, sterile pine needles were placed on the surface of SNA plates. Colony morphology and growth rates were recorded after 14–20 d incubation at 25 °C. Colony colors were described according to Rayner (1970). Micromorphological structures were examined using a Zeiss Discovery V8 stereomicroscope (Jena, Germany) for conidiomata and an Olympus BX51 compound microscope (Tokyo, Japan) for microscopic features. Structures (conidiophores, conidiogenous cells, and alpha, beta, and gamma conidia) were mounted in distilled water or lactic acid for observation and photography. For each isolate, the dimensions of 50 randomly selected conidia were measured.

### Molecular analyses

Genomic DNA was extracted from fungal mycelium harvested from colonies grown on PDA for 20 d using the Wizard® Genomic DNA Purification Kit (Promega, Madison, WI, USA), following the manufacturer’s protocol. Five partial gene regions were amplified: the internal transcribed spacer (ITS) regions, calmodulin (*cal*), histone H3 (*his3*), translation elongation factor 1-alpha (*tef1*), and beta-tubulin (*tub2*). The primer pairs used were ITS1/ITS4 for ITS (White et al. 1990), CAL-228F/CAL-737R for *cal* (Carbone and Kohn 1999), CYLH3F/H3-1b for *his3* (Glass and Donaldson 1995; Crous et al. 2004), EF1-728F/EF1-986R for *tef1* (Carbone and Kohn 1999), and T1/Bt2b for *tub2* (Glass and Donaldson 1995; O’Donnell and Cigelnik 1997). PCR amplification was performed in a 25 μL reaction volume with the following thermal cycling conditions: initial denaturation at 94 °C for 5 min; followed by 35 cycles of denaturation at 94 °C for 30 s, annealing for 50 s at temperatures specific to each locus (48 °C for ITS; 54 °C for *cal*, *tef1*, and *tub2*; 57 °C for *his3*), and extension at 72 °C for 1 min; and a final elongation at 72 °C for 7 min. PCR products were sequenced bidirectionally by Beijing Ruibo Xingke Biotechnology Co., Ltd. (Beijing, China). Consensus sequences were assembled using SeqMan v. 7.1.0 (DNASTAR Inc., Madison, WI, USA). New sequences generated in this study were deposited in GenBank (Table 1).

**Table 1.** Species of *Diaporthe* and their Genbank accession numbers.

Species	Isolates	GenBank accession numbers					References
		ITS	*cal*	*his3*	*tef1*	*tub2*	
*D. alnicola*	CFCC 70997	PQ636515	PQ635047	PQ635053	PQ635059	PQ635065	Li et al. 2025
*D. alnicola*	CFCC 70998	PQ636516	PQ635048	PQ635054	PQ635060	PQ635066	Li et al. 2025
*D. apiculata*	CGMCC 3.17533*	KP267896	NA	NA	KP267970	KP293476	Gao et al. 2016
*D. apiculata*	CFCC 53069	MK432652	MK442974	MK442999	MK578128	MK578055	Yang et al. 2021a
*D. apiculata*	LC3187	KP267866	NA	NA	KP267940	KP293446	Gao et al. 2016
*D. apiculata*	GZCC 22-0061	OP056646	NA	NA	NA	OP150642	Dissanayake et al. 2024
*D. apiculata*	**CFCC 54306**	**PX419153**	**PX438075**	**PX434710**	**PX438078**	**PX434714**	**In this study**
*D. apiculata*	**CFCC 52791**	**PX419154**	**PX438076**	**PX434711**	**PX438079**	**PX434715**	**In this study**
*D. apiculata*	**CFCC 54333**	**PX419155**	**NA**	**PX434712**	**PX438080**	**NA**	**In this study**
*D. apiculata*	**CFCC 54296**	**PX419156**	**PX438077**	**PX434713**	**PX438081**	**NA**	**In this study**
*D. arecae*	HNZZ027	MZ509555	MZ504685	MZ504696	NA	MZ504718	Yang et al. 2021b
*D. arecae*	CBS 161.64*	KC343032	KC343274	KC343516	KC343758	KC344000	Gomes et al. 2013
*D. arecae*	**CFCC 55899**	**OK339812**	**NA**	**OK358545**	**OK358562**	**OK358579**	**In this study**
*D. aseana*	MFLUCC 12-0299a*	KT459414	KT459464	NA	KT459448	KT459432	Hyde et al. 2016
*D. aseana*	MFLUCC 12-0767	KX986782	KX999284	KX999254	KX999174	KX999214	Hyde et al. 2016
*D. aseana*	LC6512	KU712429	KU749358	NA	KU749371	KU743976	Doilom et al. 2017
*D. azadirachtae*	TN 01	KC631323	NA	NA	NA	NA	Vedashree et al. 2015
*D. biguttulata*	CFCC 52584*	MH121519	MH121437	MH121477	MH121561	MH121598	Yang et al. 2018
*D. biguttulata*	CFCC 52585	MH121520	MH121438	MH121478	MH121562	MH121599	Yang et al. 2018
*D. castanopsidicola*	CFCC 54374*	**OK339805**	**OK358523**	**OK358538**	**OK358555**	**OK358572**	**In this study**
*D. castanopsidicola*	**CFCC 55908**	**OK339806**	**OK358524**	**OK358539**	**OK358556**	**OK358573**	**In this study**
*D. changjiangensis*	CFCC 54311*	**ON179822**	**NA**	**ON468713**	**ON184935**	**NA**	**In this study**
*D. changjiangensis*	**CFCC 55917**	**ON179823**	**NA**	**ON468714**	**ON184936**	**NA**	**In this study**
*D. charlesworthii*	BRIP 54884m*	KJ197288	NA	NA	KJ197250	KJ197268	Thompson et al. 2015
*D. chiangraiensis*	MFLUCC 17-1669*	MF190119	NA	NA	MF377598	NA	Senanayake et al. 2017
*D. chiangraiensis*	MFLUCC 17-1670	MF190118	NA	NA	MF377599	NA	Senanayake et al. 2017
*D. cinnamomi*	CFCC 52569*	MH121504	NA	MH121464	MH121546	MH121586	Yang et al. 2018
*D. cinnamomi*	CFCC 52570	MH121505	NA	MH121465	MH121547	MH121587	Yang et al. 2018
*D. cinnamomi*	GZCC 22-0055	OP056653	OP150650	OP150725	OP150493	OP150571	Dissanayake et al. 2024
*D. citriasiana*	ZJUD30*	JQ954645	KC357491	NA	JQ954663	KC357459	Huang et al. 2013
*D. citriasiana*	ZJUD81	KJ490616	NA	KJ490558	KJ490495	KJ490437	Huang et al. 2015
*D. cyclobalanopsidicola*	CFCC 54462*	**OK339809**	**OK358527**	**OK358542**	**OK358559**	**OK358576**	**In this study**
*D. cyclobalanopsidicola*	**CFCC 55903**	**OK339810**	**OK358528**	**OK358543**	**OK358560**	**OK358577**	**In this study**
*D. discoidispora*	ZJUD89*	KJ490624	NA	KJ490566	KJ490503	KJ490445	Huang et al. 2015
*D. discoidispora*	GZCC 22-0060	OP056658	OP150654	OP150729	OP150497	OP150575	Huang et al. 2015
*D. discoidispora*	GZCC 22-0065	OP056659	OP150655	OP150730	OP150498	OP150576	Huang et al. 2015
*D. eleutharrhenae*	01*	OK017069	NA	NA	OK017070	NA	Song and Landrein 2022
*D. eleutharrhenae*	02	OK648457	NA	NA	OK648458	OK648459	Song and Landrein 2022
*D. eres*	AR5193*	KJ210529	KJ434999	KJ420850	KJ210550	KJ420799	Udayanga et al. 2014
*D. eres*	DLR12a	KJ210518	KJ434996	KJ420833	KJ210542	KJ420783	Udayanga et al. 2014
*D. fici-septicae*	NCYUCC 19-0108*	MW114349	NA	NA	MW192212	MW148269	Tennakoon et al. 2021
*D. fici-septicae*	MFLU 20-20178	MW114348	NA	NA	MW192211	MW148268	Tennakoon et al. 2021
*D. gardeniae*	CBS 288.56*	KC343113	KC343355	KC343597	KC343839	KC344081	Gomes et al. 2013
*D. gardeniae*	step 1	KY797655	NA	MF158049	MF158048	NA	Fang et al. 2023
*D. grandiflori*	SAUCC 194.84	MT822612	MT855691	MT855580	MT855924	MT855809	Sun et al. 2021
*D. heterophyllae*	CBS 143769	MG600222	MG600218	MG600220	MG600224	MG600226	Marín-Felix et al. 2019
*D. hongkongensis*	CBS 115448*	KC343119	KC343361	KC343603	KC343845	KC344087	Gomes et al. 2013
*D. hongkongensis*	ZJUD74	KJ490609	NA	KJ490551	KJ490488	KJ490430	Huang et al. 2015
*D. hongkongensis*	BRIP 66145	MN708222	NA	NA	MN696522	MN696530	Wrona et al. 2020
*D. hongkongensis*	CGMCC 3.15175	KC153104	KF576236	NA	KC153095	KF576311	Gao et al. 2014
*D. hongkongensis*	CGMCC 3.15178	KC153103	NA	NA	KC153094	NA	Gao et al. 2014
*D. hongkongensis*	CFCC 53101	MK432643	MK442965	MK442990	MK578119	MK578046	Cao et al. 2022
*D. hongkongensis*	CFCC 53102	MK432644	MK442966	MK442991	MK578120	MK578047	Cao et al. 2022
*D. hongkongensis*	MFLUCC 18-0553	MN047098	NA	NA	MN077073	NA	Dayarathne et al. 2020
*D. hongkongensis*	MFLU 17-2592	MN047099	NA	NA	MN077074	NA	Dayarathne et al. 2020
*D. irregularis*	CGMCC 3.20092*	MT385951	MT424721	NA	MT424686	MT424706	Dissanayake et al. 2020
*D. irregularis*	GZCC 19-0344	MT797179	MT786249	NA	MT793022	MT793033	Dissanayake et al. 2020
*D. kyushuensis*	STE-U2675*	AF230749	NA	NA	NA	NA	Zheng et al. 2025
*D. kyushuensis*	ch-D-1	AB302250	NA	NA	NA	NA	Zheng et al. 2025
*D. liangxii*	CFCC 54324*	**ON179811**	**ON184913**	**ON468702**	**ON184924**	**ON184937**	**In this study**
*D. liangxii*	**CFCC 55901**	**ON179812**	**ON184914**	**ON468703**	**ON184925**	**ON184938**	**In this study**
*D. linzhiensis*	CFCC 71057*	PQ636519	PQ635051	PQ635057	PQ635063	PQ635069	Li et al. 2025
*D. linzhiensis*	N266C	PQ636520	PQ635052	PQ635058	PQ635064	PQ635070	Li et al. 2025
*D. lithocarpicola*	CFCC 54297*	**OK339815**	**OK358531**	**OK358548**	**OK358565**	**OK358582**	**In this study**
*D. lithocarpicola*	**CFCC 55913**	**OK339816**	**OK358532**	**OK358549**	**OK358566**	**OK358583**	**In this study**
*D. oxe*	CBS 133186*	KC343164	KC343406	KC343648	KC343890	KC344132	Gomes et al. 2013
*D. oxe*	CBS 133187	KC343165	KC343407	KC343649	KC343891	KC344133	Gomes et al. 2013
*D. penetriteum*	LC3353	KP714505	NA	KP714493	KP714517	KP714529	Gao et al. 2016
*D. quercicola*	CSUFTCC104*	ON076567	ON081670	ON081667	ON081659	NA	Cao et al. 2022
*D. quercicola*	CSUFTCC105	ON076568	ON081671	ON081668	ON081660	NA	Cao et al. 2022
*D. sennicola*	CFCC 51634*	KY203722	KY228873	KY228879	KY228883	KY228889	Yang et al. 2017
*D. sennicola*	CFCC 51635	KY203723	KY228874	KY228880	KY228884	KY228890	Yang et al. 2017
*D. shennongjiaensis*	CNUCC 201905*	MN216229	MN224551	MN224559	MN224672	MN227012	Zhou and Hou 2019
*D. shennongjiaensis*	CNUCC 201906	MN216228	MN224552	MN224561	MN224673	MN227013	Zhou and Hou 2019
*D. siamensis*	MFLUCC 10-0573a*	JQ619879	JX197423	NA	JX275393	JX275429	Udayanga et al. 2012
*D. siamensis*	MFLUCC 12-0300	KT459417	KT459467	NA	KT459451	KT459435	Udayanga et al. 2012
*D. siamensis*	**CFCC 54817**	**OK339819**	**OK358535**	**OK358552**	**OK358569**	**OK358586**	**In this study**
*D. siamensis*	**CFCC 55916**	**OK339820**	**OK358536**	**OK358553**	**OK358570**	**OK358587**	**In this study**
*D. siamensis*	**CFCC 54295**	**OK339821**	**OK358537**	**OK358554**	**OK358571**	**OK358588**	**In this study**
*D. virgiliae*	CMW 40755	KP247573	NA	NA	NA	KP247582	Machingambi et al. 2015
*D. virgiliae*	CMW 40748	KP247566	NA	NA	NA	KP247575	Machingambi et al. 2015
*D. xishuangbanica*	CGMCC 3.18282	KX986783	NA	KX999255	KX999175	KX999216	Gao et al. 2017
*D. yunnanensis*	CGMCC 3.18289*	KX986796	KX999290	KX999267	KX999188	KX999228	Gao et al. 2017
*D. yunnanensis*	LC8107	KY491542	KY491572	NA	KY491552	KY491562	Gao et al. 2017
*D. zaofenghuang*	CGMCC 3.20271	MW477883	MW480867	MW480863	MW480871	MW480875	Wang et al. 2021
*D. zaofenghuang*	TZFH3	MW477884	MW480868	MW480864	MW480872	MW480876	Wang et al. 2021
*D. zhaoqingensis*	ZHKUCC 22-0056*	ON322885	ON315000	ON315015	NA	ON315074	Luo et al. 2022
*D. zhaoqingensis*	ZHKUCC 22-0057	ON322886	NA	ON315016	ON315043	ON315075	Luo et al. 2022

Note. Ex-type strains are marked with *, and NA means not available.

Sequence alignments for individual loci were generated using MAFFT v. 7 (Katoh and Standley 2013) and manually adjusted in MEGA v. 7.0.21 where necessary. Based on BLASTn searches of ITS sequences, isolates were assigned to their respective species complexes (*D. arecae*, *D. gardeniae*, *D. siamensis*, and *D. virgiliae* SCs) for further detailed analyses. Phylogenetic analyses were performed on concatenated five-locus datasets (ITS-*cal*-*his3*-*tef1*-*tub2*) using both Maximum Likelihood (ML) and Bayesian Inference (BI) approaches implemented in the One-click Fungal Phylogenetic Tool (OFPT) v. 1.9.0 (Zeng et al. 2023). For ML analysis, the best-fit substitution models were determined using ModelFinder (Kalyaanamoorthy et al. 2017). ML trees were inferred using IQ-TREE with ultrafast bootstrap approximation (1000 replicates) (Minh et al. 2020; Hoang et al. 2018). Bayesian Inference was performed using MrBayes v. 3.2.7 (Ronquist et al. 2012), with best-fit models selected by ModelFinder under the Akaike Information Criterion (AIC). Two parallel runs of four Markov chains (one cold, three heated) were executed for 10 million generations, sampling every 100 generations. The first 25% of sampled trees were discarded as burn-in, and the remaining trees were used to calculate posterior probabilities (PP). Convergence was confirmed when the average standard deviation of split frequencies fell below 0.01. Phylogenetic trees were visualized in FigTree v. 1.4.4 (Rambaut 2018).

To evaluate the genealogical exclusivity of the novel lineages, the Pairwise Homoplasy Index (PHI) test was conducted in SplitsTree4 v. 4.14.6 (Huson and Bryant 2006) to detect recombination events (Φw). A *p*-value > 0.05 indicates a lack of significant recombination. Additionally, species boundaries were assessed using two coalescence-based methods: the Poisson Tree Processes (PTP) model and the Multi-rate PTP (mPTP) model. Analyses were conducted on the respective web servers (http://species.h-its.org/ptp/ and http://mptp.h-its.org/) using the ML tree as input, with 1,000,000 MCMC generations, a thinning of 100, and a burn-in of 10% (Zhang et al. 2013; Kapli et al. 2017).

## Results

### Phylogeny

**Phylogenetic analyses of the *Diaporthe arecae* species complex**

**Figure 1.**
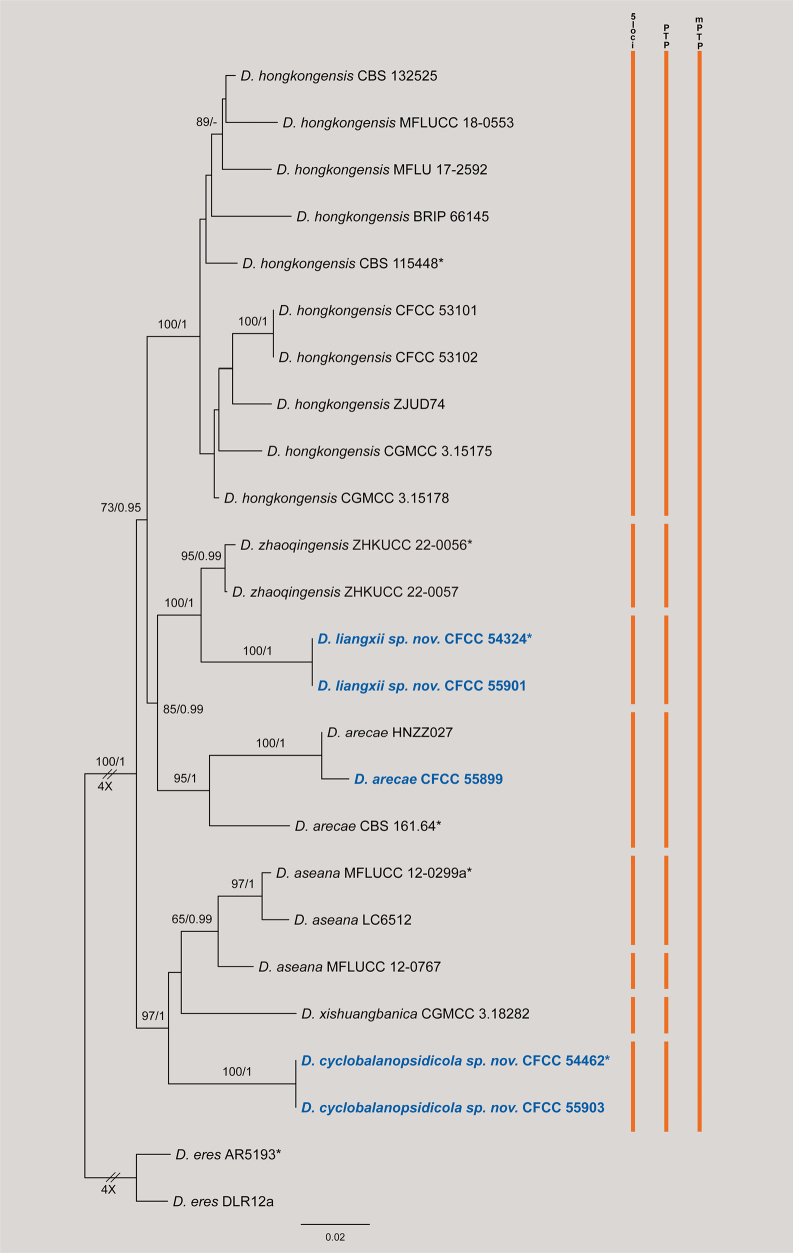
Phylogram of *Diaporthe arecae* and related species resulting from a maximum likelihood analysis based on the ITS, *cal*, *his3*, *tef1* and *tub2* gene loci. Numbers above the branches indicate ML bootstrap values (left, MLBS ≥ 50%) and Bayesian posterior probabilities (right, BPP ≥ 0.90). Ex-type strains are marked with *, and isolates from this study are in bold blue. Colored vertical bars on the right indicate species delimitation results based on the 5-locus phylogeny (5-loci), Poisson Tree Processes (PTP), and Multi-rate PTP (mPTP). The scale bar represents the expected number of nucleotide substitutions per site. The tree is rooted with *D. eres* (AR5193 and DLR12a).

The concatenated dataset of five loci (ITS, *cal*, *his3*, *tef1*, and *tub2*) for the *Diaporthe arecae* species complex comprised 25 strains, including *D. eres* (AR5193 and DLR12a) as the outgroup. The alignment consisted of 2,581 characters (including gaps) partitioned as follows: *cal* (1–485), *his3* (486–949), ITS (950–1483), *tef1* (1484–1807), and *tub2* (1808–2581). The alignment contained 640 distinct site patterns, with 30.49% of characters consisting of gaps or missing data. The best-fit substitution models were estimated as TNe+R2 for ITS, TNe+G4 for *cal*, HKY+F+I for *his3*, and HKY+F+G4 for both *tef1* and *tub2*. The Maximum Likelihood (ML) analysis yielded a best tree with a log-likelihood value of –8485.05. The estimated gamma shape parameter (*α*) was 0.262. Bayesian Inference (BI) resulted in a topology congruent with the ML tree; thus, only the ML tree is presented with statistical support values (ML-BS/BI-PP) annotated at the nodes (Fig. 1).

In the phylogenetic tree, the isolates obtained in this study were resolved into three distinct lineages. (1) Two strains (CFCC 54324 and CFCC 55901) formed a highly supported clade (ML-BS/BI-PP = 100/1), described herein as *D. liangxii*. This species formed a sister lineage to *D. zhaoqingensis* (95/0.99). (2) Two strains (CFCC 54462 and CFCC 55903) formed a distinct, fully supported clade (100/1), described herein as *D. cyclobalanopsidicola*. This species appeared as a sister lineage to the clade containing *D. aseana* and *D. xishuangbanica*. (3) Isolate CFCC 55899 clustered within the *D. arecae* clade with strong support (100/1).

Species delimitation analyses yielded slightly conflicting results. The PTP analysis (Fig. 1) recognized *D. liangxii* and *D. cyclobalanopsidicola* as independent evolutionary lineages. However, the multi-rate PTP (mPTP) analysis was more conservative, delimiting *D. liangxii* and *D. zhaoqingensis* as a single molecular entity, and similarly merging *D. cyclobalanopsidicola* with *D. aseana* and *D. xishuangbanica*. Despite the mPTP results, distinct morphological traits and significant genetic distances (in *tef1* and *tub2*) support their status as distinct species.

#### Phylogenetic analyses of the *Diaporthe gardeniae* species complex

**Figure 2.**
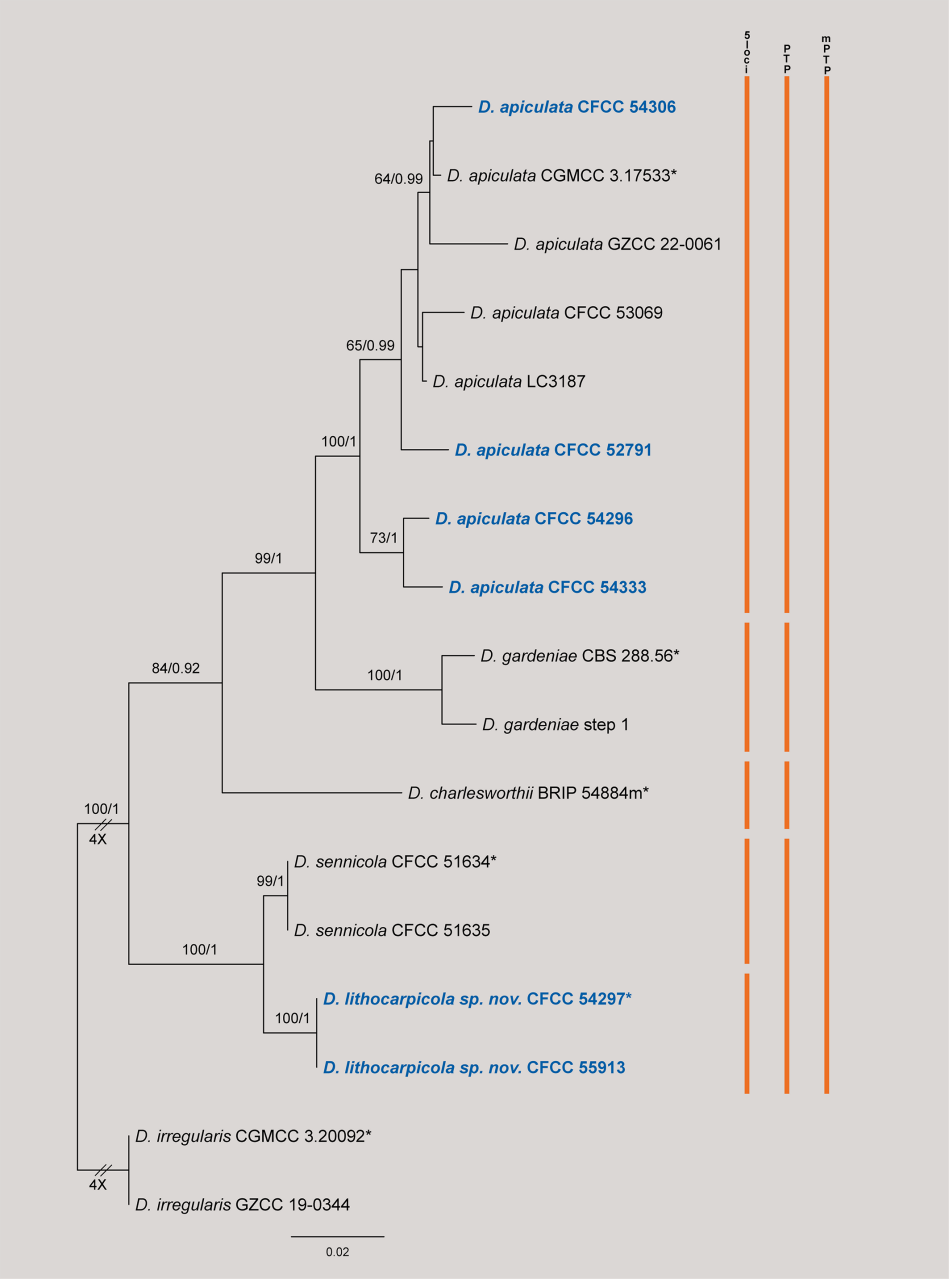
Phylogram of *Diaporthe gardeniae* species complex resulting from a maximum likelihood analysis based on the ITS, *cal*, *his3*, *tef1* and *tub2* gene loci. Numbers above the branches indicate ML bootstrap values (left, MLBS ≥ 50%) and Bayesian posterior probabilities (right, BPP ≥ 0.90). Ex-type strains are marked with *, and isolates from this study are in bold blue. Colored vertical bars on the right indicate species delimitation results based on the 5-locus phylogeny (5-loci), Poisson Tree Processes (PTP), and Multi-rate PTP (mPTP). The scale bar represents the expected number of nucleotide substitutions per site. The tree is rooted with *D. irregularis* (CGMCC 3.20092 and GZCC 19-0344).

The concatenated dataset (ITS, *cal*, *his3*, *tef1*, and *tub2*) for the *Diaporthe gardeniae* species complex comprised 18 strains, with *D. irregularis* (CGMCC 3.20092 and GZCC 19-0344) serving as the outgroup. The alignment contained 2,113 characters (including gaps) partitioned as follows: *cal* (1–469), *his3* (470–929), ITS (930–1387), *tef1* (1388–1719), and *tub2* (1720–2113). The alignment comprised 372 distinct site patterns, with 24.58% gaps or missing data. Model selection utilizing ModelFinder determined the best-fit substitution models as K2P+R2 for ITS, K2P+G4 for *cal*, TN+F+G4 for *his3*, TIM2e+G4 for *tef1*, and HKY+F+I for *tub2*. The ML analysis yielded a best tree with a log-likelihood value of –5761.24, and the estimated gamma shape parameter was 0.183. Bayesian inference produced a topology consistent with the ML tree (Fig. 2).

In the phylogenetic tree, the isolates from this study clustered into two groups: (1) Four isolates (CFCC 54306, CFCC 52791, CFCC 54296, and CFCC 54333) clustered within the *D. apiculata* clade with strong statistical support (BS/PP = 100/1). (2) Two isolates (CFCC 54297 and CFCC 55913) formed a distinct, fully supported clade (100/1), described herein as *D. lithocarpicola*. This species formed a sister lineage to *D. sennicola* (CFCC 51634 and CFCC 51635).

Both PTP and mPTP analyses lumped *D. lithocarpicola* and *D. sennicola* into a single evolutionary entity (Fig. 2). However, these two taxa formed well-supported, distinct clades in the multi-locus phylogeny (100% BS / 1.00 PP). Furthermore, a comparison of nucleotide sequences revealed significant genetic distinctions. For instance, *D. lithocarpicola* differs from *D. sennicola* by 13 base pairs (bp) in *cal*, 2 base pairs (bp) in *his3*, 14 base pairs (bp) in *tef1* and 5 bp in *tub2*. Coupled with distinct morphological characteristics, we strictly defined *D. lithocarpicola* as a novel species following the GCPSR concept.

#### Phylogenetic analyses of the *Diaporthe siamensis* species complex

**Figure 3.**
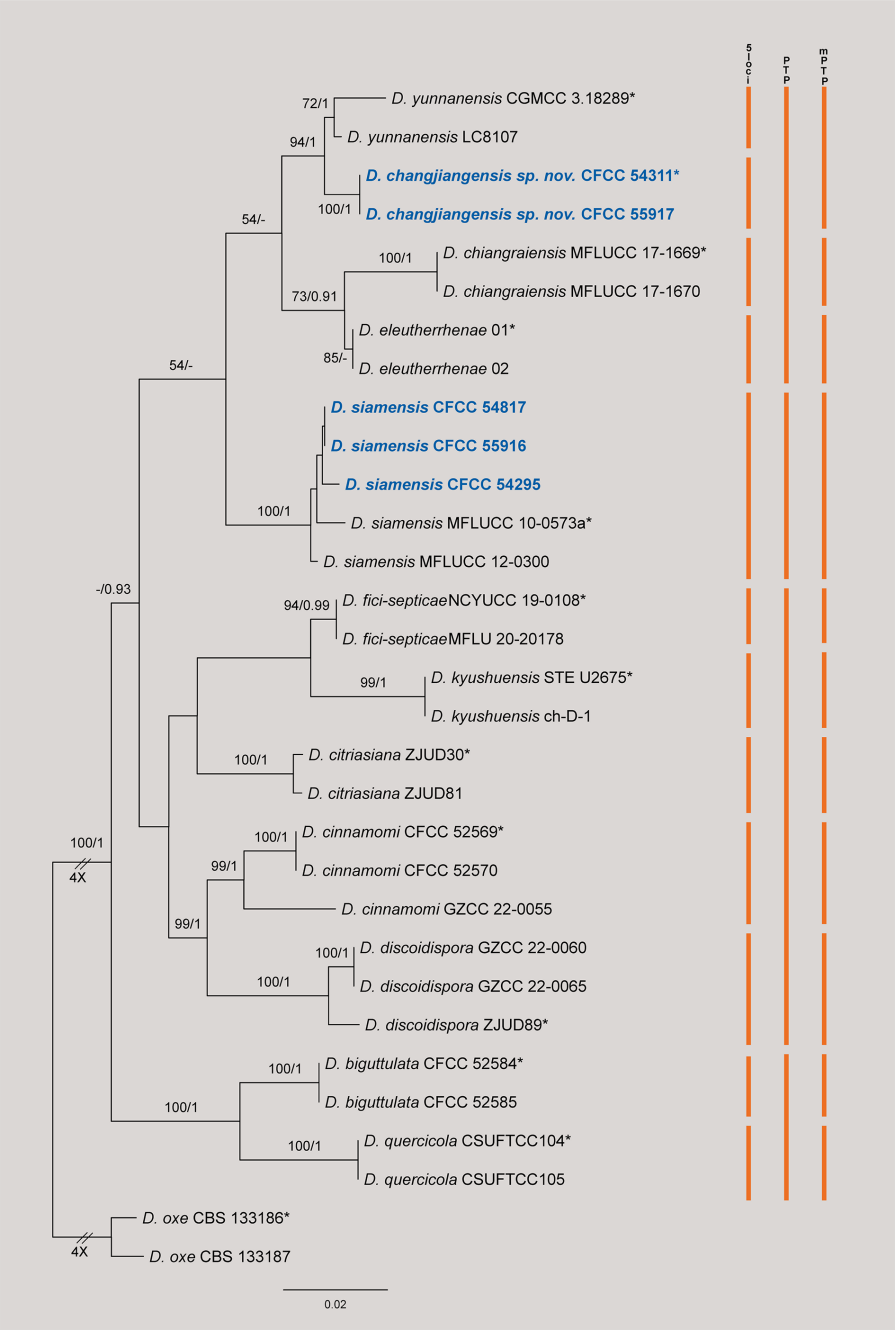
Phylogram of *Diaporthe siamensis* species complex resulting from a maximum likelihood analysis based on the ITS, *cal*, *his3*, *tef1* and *tub2* gene loci. Numbers above the branches indicate ML bootstrap values (left, MLBS ≥ 50%) and Bayesian posterior probabilities (right, BPP ≥ 0.90). Ex-type strains are marked with *, and isolates from this study are in bold blue. Colored vertical bars on the right indicate species delimitation results based on the 5-locus phylogeny (5-loci), Poisson Tree Processes (PTP), and Multi-rate PTP (mPTP). The scale bar represents the expected number of nucleotide substitutions per site. The tree is rooted with *D. oxe* (CBS 133186 and CBS 133187).

The concatenated dataset (ITS, *cal*, *his3*, *tef1*, and *tub2*) for the *Diaporthe siamensis* species complex comprised 31 strains, rooted with *D. oxe* (CBS 133186 and CBS 133187). The alignment consisted of 2,460 characters (including gaps) partitioned as follows: *cal* (1–452), *his3* (453–902), ITS (903–1360), *tef1* (1361–1690), and *tub2* (1691–2460). The matrix contained 531 distinct site patterns, with 33.83% of characters consisting of gaps or missing data. Model selection identified the best-fit substitution models as K2P+R2 for ITS, K2P+G4 for *cal*, TN+F+I for *his3*, TIM2e+G4 for *tef1*, and HKY+F+G4 for *tub2*. The ML analysis resulted in a best tree with a log-likelihood value of –7544.83, and the estimated gamma shape parameter (*α*) was 0.229. Bayesian Inference (BI) recovered a topology congruent with the ML tree (Fig. 3).

In the phylogenetic tree, the isolates obtained in this study were assigned to two lineages: (1) Three isolates (CFCC 54817, CFCC 55916, and CFCC 54295) clustered with the ex-type strain of *D. siamensis* (MFLUCC 10-0573a) with maximum support (BS/PP = 100/1). (2) Two isolates (CFCC 54311 and CFCC 55917) formed a distinct, highly-supported clade (100/1), described herein as *D. changjiangensis*. This species resides in a sister relationship with *D. yunnanensis* (CGMCC 3.18289 and LC8107), separated by high statistical support (94/0.99).

Similar to the *D. gardeniae* complex, species delimitation analyses for this group yielded results incongruent with the phylogeny. Both PTP and mPTP analyses suggested that *D. changjiangensis* and *D. yunnanensis* constitute a single species (Fig. 3). However, *D. changjiangensis* differs from *D. yunnanensis* by 23 bp in ITS, 9 bp in *his3*, and 3 bp in *tef1*. Based on these genetic distinctions and morphological differences, we recognize *D. changjiangensis* as a novel species.

#### Phylogenetic analyses of the *Diaporthe virgiliae* species complex

**Figure 4.**
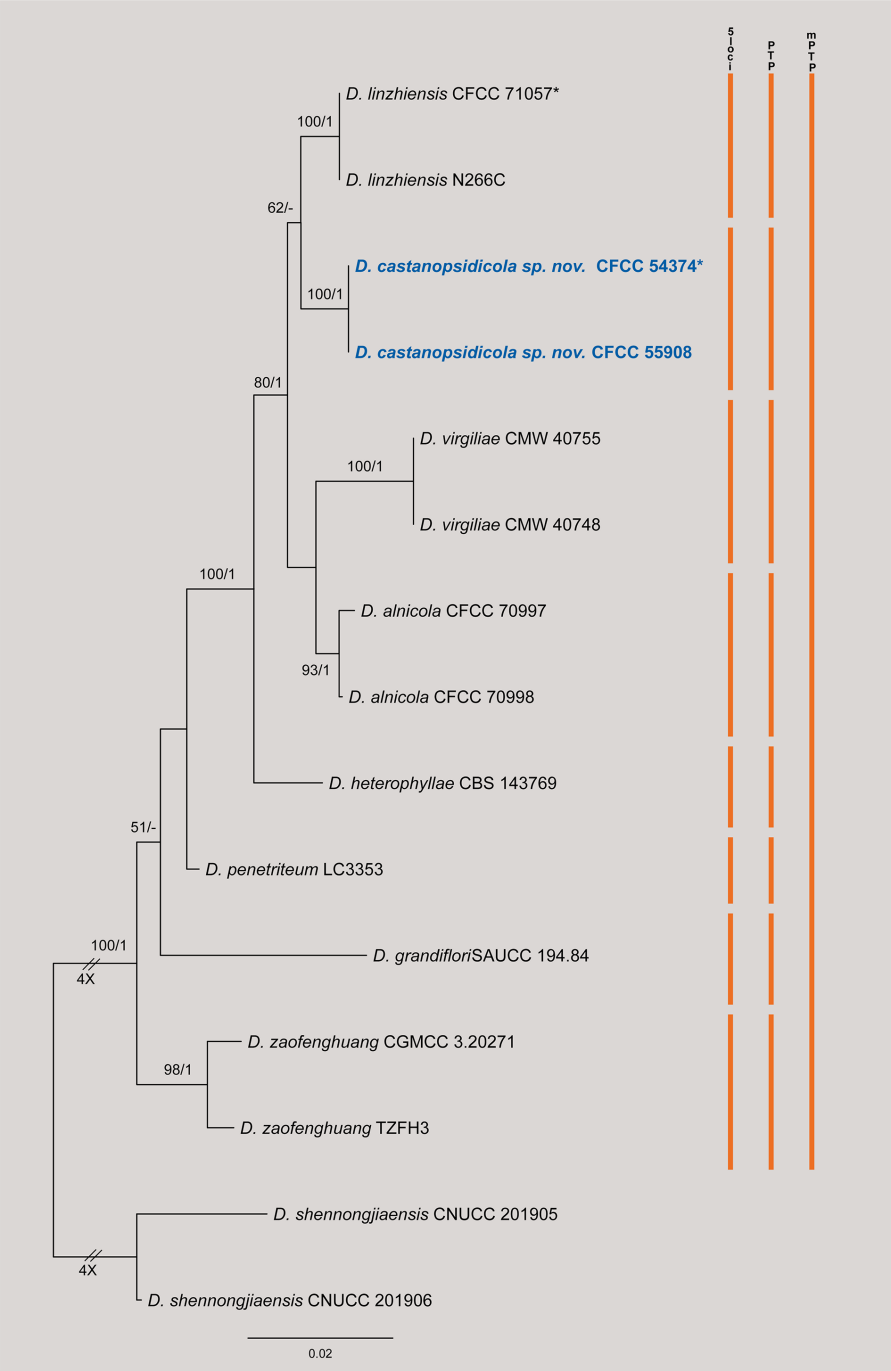
Phylogram of *Diaporthe virgiliae* species complex resulting from a maximum likelihood analysis based on the ITS, *cal*, *his3*, *tef1* and *tub2* gene loci. Numbers above the branches indicate ML bootstrap values (left, MLBS ≥ 50%) and Bayesian posterior probabilities (right, BPP ≥ 0.90). Ex-type strains are marked with *, and isolates from this study are in bold blue. The scale bar represents the expected number of nucleotide substitutions per site. Colored vertical bars on the right indicate species delimitation results based on the 5-locus phylogeny (5-loci), Poisson Tree Processes (PTP), and Multi-rate PTP (mPTP). The tree is rooted with *D. shennongjiaensis* (CNUCC 201905 and CNUCC 201906).

The concatenated dataset (ITS, *cal*, *his3*, *tef1*, and *tub2*) for the *Diaporthe virgiliae* species complex comprised 15 strains, rooted with *D. shennongjiaensis* (CNUCC 201905 and CNUCC 201906). The alignment consisted of 2,585 characters (including gaps) partitioned as follows: *cal* (1–413), *his3* (414–879), ITS (880–1472), *tef1* (1473–1798), and *tub2* (1799–2585). The matrix contained 348 distinct site patterns, with 19.27% of characters comprising gaps or missing data. Model selection identified the best-fit substitution models as K2P+I for ITS, K2P for *cal*, TN+F+I for *his3*, HKY+F+R2 for *tef1*, and HKY+F+I for *tub2*. The ML analysis resulted in a best tree with a log-likelihood value of –5750.79, and the estimated gamma shape parameter (*α*) was 0.02. Bayesian Inference (BI) recovered a topology congruent with the ML tree (Fig. 4).

In the phylogenetic tree, the two isolates obtained in this study (CFCC 54374 and CFCC 55908) formed a highly supported, distinct clade (BS/PP = 100/1), described herein as *D. castanopsidicola*. Phylogenetically, *D. castanopsidicola* is sister to *D. linzhiensis* (CFCC 71057 and N266C).

Species delimitation analyses yielded remarkably distinct results. The PTP analysis successfully delimited *D. castanopsidicola* as an independent evolutionary lineage separate from *D. linzhiensis* (Fig. 4). In contrast, the mPTP analysis produced a highly conservative delimitation, lumping *D. castanopsidicola* into a large species aggregate that includes *D. linzhiensis*, *D. virgiliae*, *D. alnicola*, and *D. heterophyllae*. Given that *D. virgiliae* and *D. heterophyllae* are well-established biological species, the lumping result of mPTP is considered an artifact of the method’s conservatism in this dataset. Therefore, based on the robust phylogenetic separation (100% BS) and morphological distinctions, we establish *D. castanopsidicola* as a novel species.

### Evaluation of recombination (PHI analysis)

**Figure 5.**
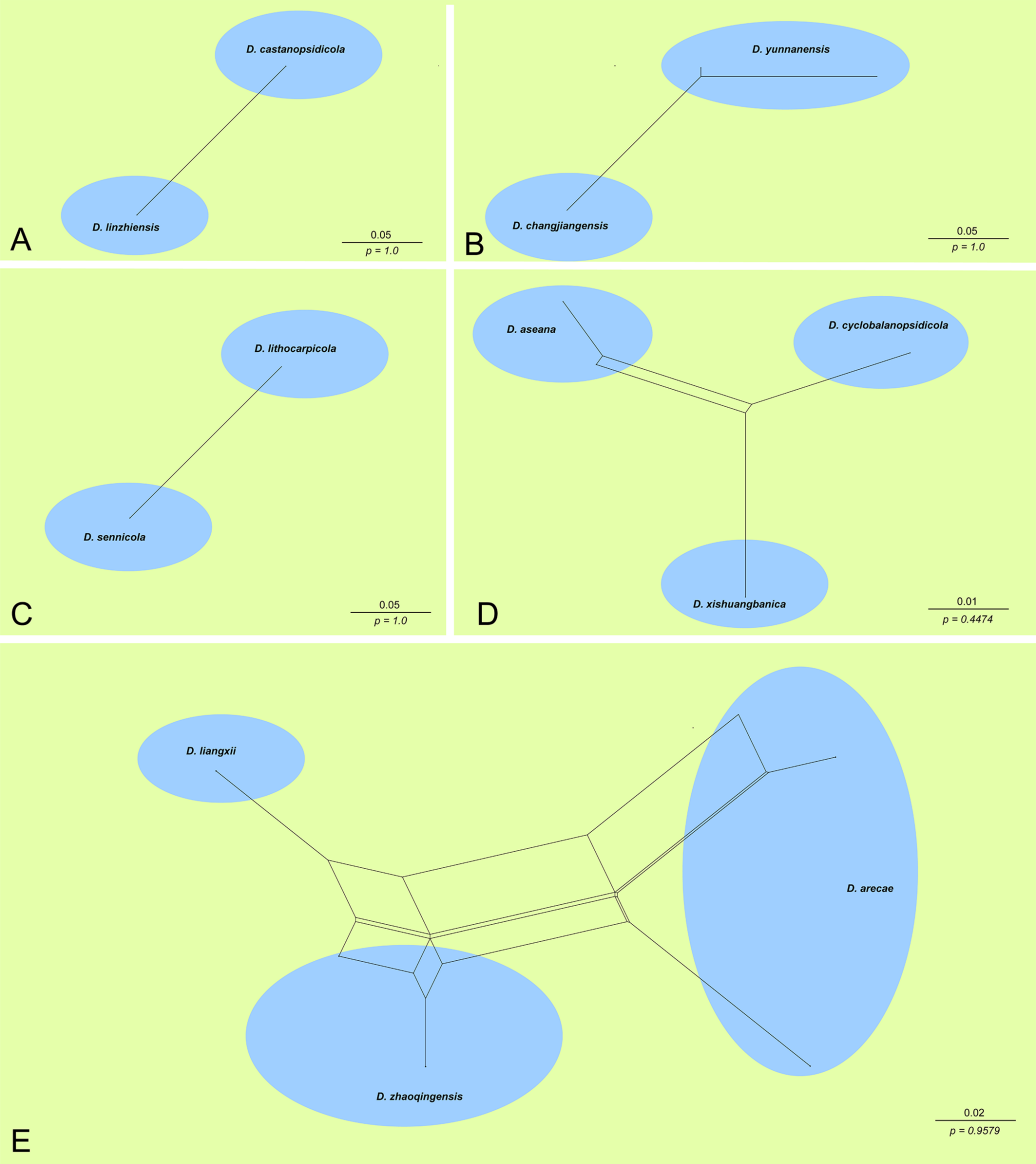
The split graphs of a PHI test result of *Diaporthe* species using the LogDet transformation and split decomposition options based on the combined ITS, *cal*, *his3*, *tef1* and *tub2* gene loci. **A***p* = 1.0. **B***p* = 1.0. **C***p* = 1.0. **D***p* = 0.4474. **E***p* = 0.9579.

To further validate the genealogical exclusivity of the novel lineages, the Pairwise Homoplasy Index (PHI) test was performed to detect potential recombination events between the new species and their phylogenetically closest relatives. Five specific datasets were constructed for assessment: (A) *D. castanopsidicola* vs. *D. linzhiensis*; (B) *D. changjiangensis* vs. *D. yunnanensis*; (C) *D. lithocarpicola* vs. *D. sennicola*; (D) *D. cyclobalanopsidicola* vs. *D. aseana* and *D. xishuangbanica*; and (E) *D. liangxii* vs. *D. zhaoqingensis* and *D. arecae*. The PHI test results revealed no statistically significant evidence of genetic recombination (*p* > 0.05) within any of the tested groups (Fig. 5). Specifically, comparisons for Clades A, B, and C yielded a *p* value of 1.0, indicating a lack of recombination signal. Similarly, Clades D (*p* = 0.447) and E (*p* = 0.958) showed no significant recombination. These findings confirm the absence of gene flow between the novel species and their sister taxa, reinforcing their validity as distinct evolutionary lineages.

### Taxonomy

#### Diaporthe apiculata

Taxon classificationAnimaliaDiaporthalesDiaporthaceae

Y.H. Gao, F. Liu & L. Cai, Syst. Biodivers. 14: 106 (2016)

0E95B2B8-1934-5002-8614-6D096E8F9295

Fig. 6

##### Description.

See Gao et al. (2016).

##### Distribution.

China.

##### Ecology.

Associated with leaf spot disease of *Castanopsis chunii*, *C. hystrix*, *Lithocarpus amygdalifolius* and *L. naiadarum*, branch canker disease of *Rhus chinensis*, and endophytic in *Camellia sinensis*.

##### Materials examined.

CHINA • Hainan Province, Changjiang Li Autonomous County, Bawangling National Forest Park, on diseased leaves of *Lithocarpus naiadarum*, 24 November 2019, Yong Li (culture CFCC 52791); • Guangdong Province, Qingyuan City, Yangshan County, Guangdong Nanling Nature Reserve, on diseased leaves of *Castanopsis chunii*, 26 November 2019, Dan-ran Bian (culture CFCC 54306); • Hainan Province, Changjiang Li Autonomous County, Bawangling National Forest Park, on diseased leaves of *Lithocarpus amygdalifolius*, 31 March 2019, Yong Li (culture CFCC 54333); • Hainan Province, Changjiang Li Autonomous County, Bawangling National Forest Park, on diseased leaves of *Castanopsis hystrix*, 31 March 2019, Yong Li (culture CFCC 54296).

**Figure 6.**
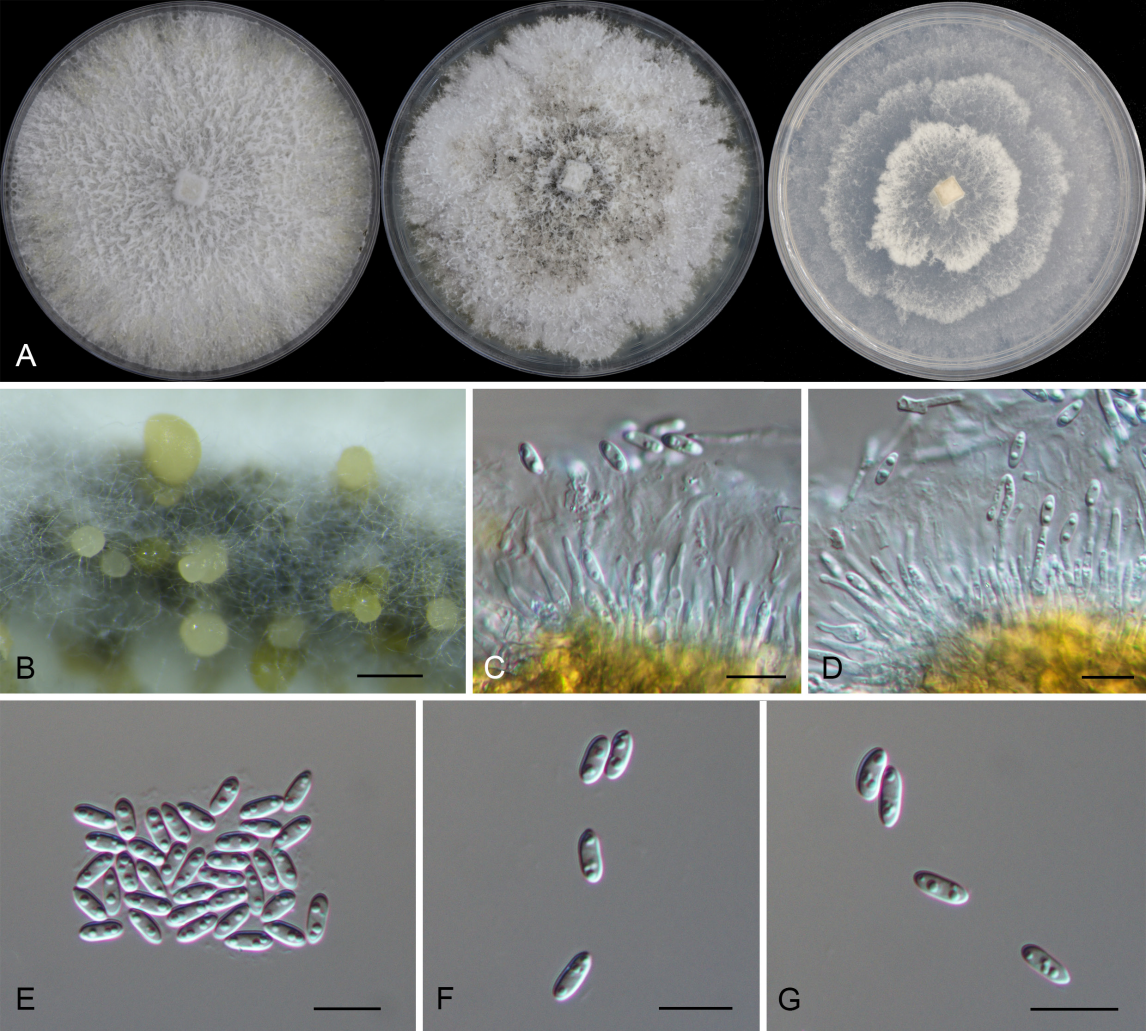
Morphology of *Diaporthe apiculata* (CFCC 52791). **A** Colonies on PDA, MPA and SNA after 20 d at 25 °C. **B** Conidiomata. **C, D** Conidiogenous cells giving rise to conidia. **E–G** Alpha conidia. Scale bars: 200 μm (**B**); 10 μm (**C–G**).

##### Notes.

*Diaporthe apiculata* was originally described as an endophyte from *Camellia sinensis* in China (Gao et al. 2016) and subsequently reported causing branch cankers on *Rhus chinensis* (Yang et al. 2021a). In the present study, four isolates were recovered from leaf spots of *Castanopsis chunii*, *C. hystrix*, *Lithocarpus amygdalifolius*, and *L. naiadarum*. Phylogenetically, these isolates formed a highly supported clade (ML/BI = 100/1) with the ex-type strain of *D. apiculata* (CGMCC 3.17533) (Fig. 2). To our knowledge, this is the first report of *D. apiculata* associated with *Castanopsis* and *Lithocarpus* species, expanding the known host range of this fungus to *Fagaceae*.

#### Diaporthe arecae

Taxon classificationAnimaliaDiaporthalesDiaporthaceae

(H.C. Srivast., Zakia & Govindar.) R.R. Gomes, Glienke & Crous, Persoonia 31: 16 (2013)

BAD6FC2C-9D00-509C-874D-3E9E01EB9438

Fig. 7

##### Description.

***Conidiomata*** pycnidial, aggregated or solitary, erumpent, subglobose, black, 300–1200 μm diam., exuding white conidial masses. ***Conidiophores*** indistinct, usually reduced to conidiogenous cells. ***Conidiogenous cells*** hyaline, smooth, cylindrical to ampulliform, attenuate towards the apex, phialidic, 10.5–28.5 × 1.5–3.5 μm. ***Alpha conidia*** aseptate, hyaline, smooth, multi-guttulate, fusoid, straight or slightly curved, base truncate, (5.5–)6.5–8.5(–9.5) × 2–2.5(–3) μm (n = 50), L/W = 2.2–4.6. ***Beta conidia*** aseptate, hyaline, smooth, guttulate or not, filiform, curved or not, (12–)13.5–19.5(–24.5) × 1.5–2 μm (n = 50), L/W = 5.7–15.5.

**Figure 7.**
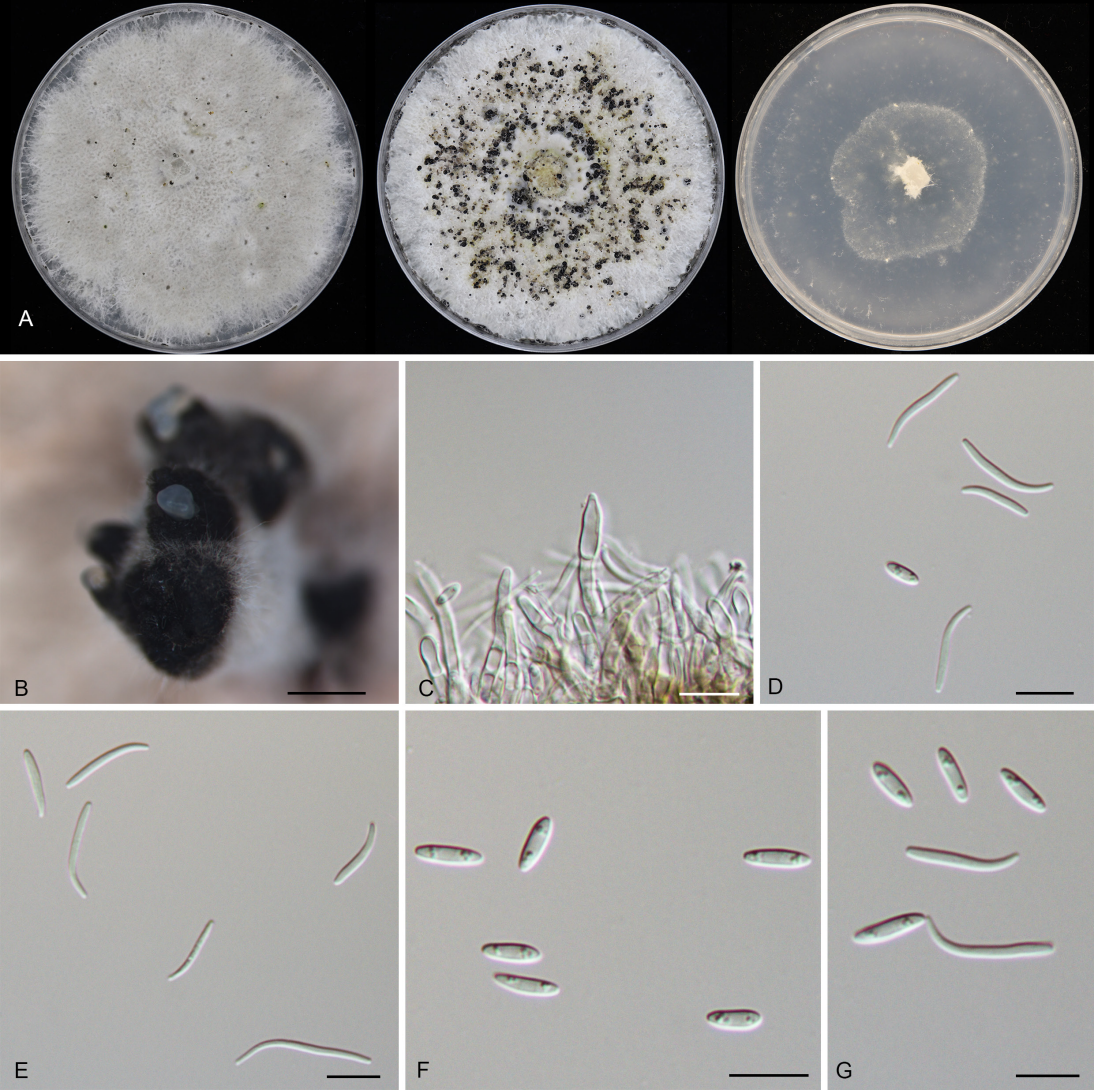
Morphology of *Diaporthe arecae* (CFCC 55899). **A** Colonies on PDA, MPA and SNA after 20 d at 25 °C. **B** Conidiomata. **C** Conidiogenous cells. **D–G** Alpha and beta conidia. Scale bars: 400 μm (**B**); 10 μm (**C–G**).

##### Culture characteristics.

Colonies on PDA flat, spreading, flocculent, with entire edge, white to mouse grey, reaching 25 mm in diam. after 10 days at 25 °C; colonies on MPA flat, spreading, flocculent, with entire edge, white, reaching 25 mm in diam. after 10 days at 25 °C, forming black conidiomata with off-white masses; colonies on SNA flat, with sparse aerial mycelium and entire margin, white, reaching 25 mm in diam. after 20 days at 25 °C.

##### Distribution.

Worldwide.

##### Ecology.

Associated with leaf spot, branch canker and fruit rot diseases on various hosts, including *Citrus* sp., *Cyclobalanopsis glauca*, *Musa* sp., *Pterocarpus indicus*, *Senna bicapsularis*, *Subramanella arecae*, etc.

##### Materials examined.

CHINA • Hainan Province, Changjiang Li Autonomous County, Bawangling National Forest Park, on diseased leaves of *Cyclobalanopsis glauca*, 30 March 2019, Yong Li (culture CFCC 55899).

##### Notes.

*Diaporthe arecae* was recently circumscribed based on a combination of phylogenetic analysis, GCPSR, and coalescence-based models (Dissanayake et al. 2024). In the current study, isolate CFCC 55899 clustered with the ex-type strain of *D. arecae* (CBS 161.64) in the multi-locus phylogeny (Fig. 1). To our knowledge, this is the first report of *D. arecae* causing leaf spots on *Cyclobalanopsis glauca* in China.

#### Diaporthe castanopsidicola

Taxon classificationAnimaliaDiaporthalesDiaporthaceae

Ning Jiang sp. nov.

D6502957-32AA-59F3-9B9B-41868CFB6B64

841314

Fig. 8

##### Etymology.

Referring to the host genus, *Castanopsis*, from which the holotype was isolated.

##### Diagnosis.

Distinct from its phylogenetically related species *D. linzhiensis* by larger conidiomata.

##### Typus.

CHINA • Guangdong Province, Qingyuan City, Yangshan County, Guangdong Nanling Nature Reserve, on diseased leaves of *Castanopsis chunii*, 26 November 2019, Dan-ran Bian (holotype CAF800027; ex-holotype culture CFCC 54374).

**Figure 8.**
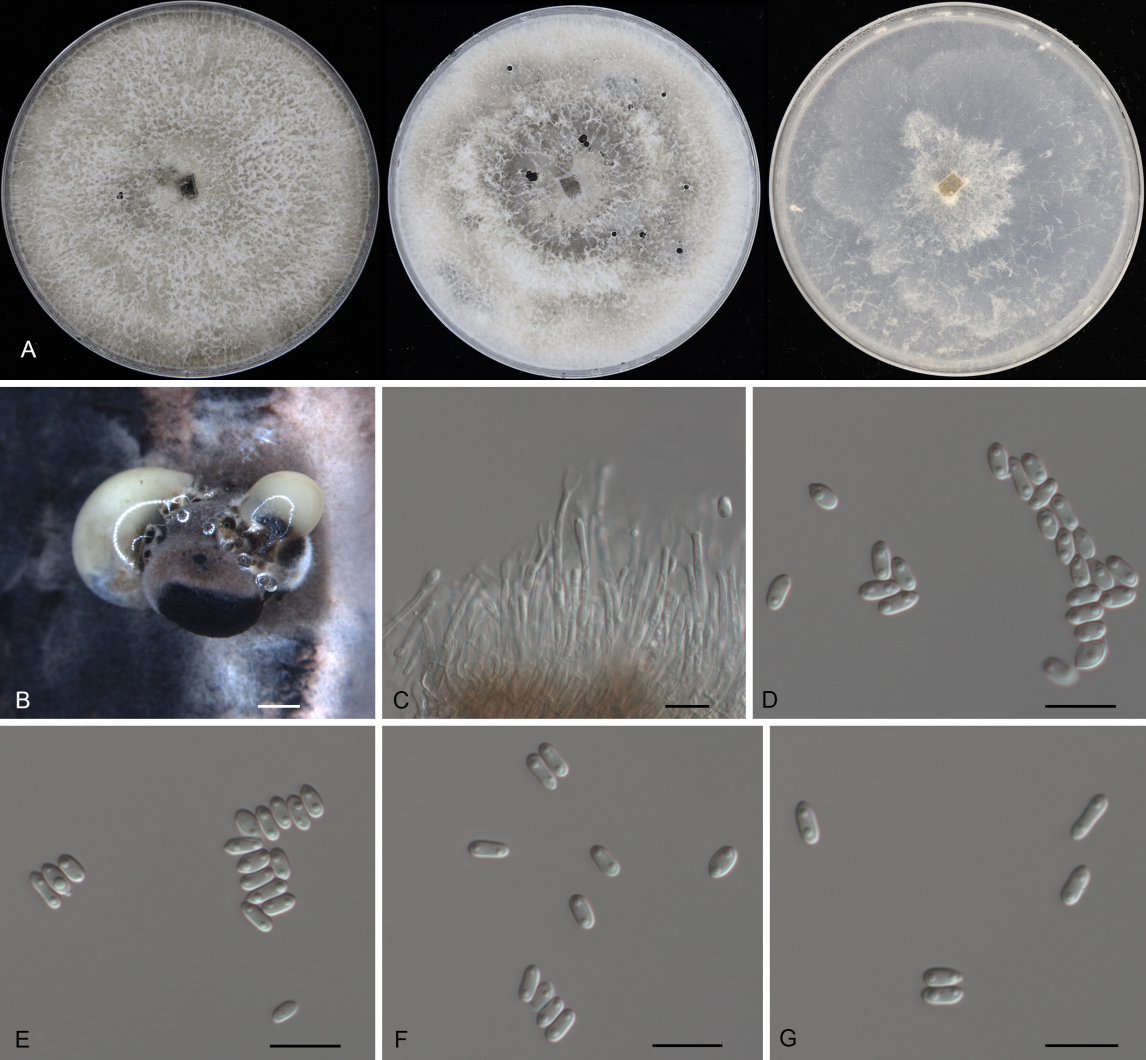
Morphology of *Diaporthe castanopsidicola* (CFCC 54374). **A** Colonies on PDA, MPA and SNA after 20 d at 25 °C. **B** Conidiomata. **C** Conidiogenous cells giving rise to conidia. **D–G** Alpha conidia. Scale bars: 400 μm (**B**); 10 μm (**C–G**).

##### Description.

***Conidiomata*** pycnidial, aggregated, erumpent, pulvinate, subglobose, umber, 800–2000 μm diam. ***Conidiophores*** indistinct, usually reduced to conidiogenous cells. ***Conidiogenous cells*** hyaline, smooth, cylindrical to filiform, phialidic, 19.5–41.5 × 1.5–3.5 μm. ***Alpha conidia*** aseptate, hyaline, smooth, biguttulate, cylindrical, slightly constricted at the middle, straight, base truncate, (4.5–)5–6(–6.5) × (2–)2.5–3 μm (n = 50), L/W = 1.6–2.6.

##### Culture characteristics.

Colonies on PDA flat, spreading, flocculent, with entire edge, initially white, becoming mouse grey with age, reaching 25 mm in diam. after 10 days at 25 °C; colonies on MPA flat, spreading, flocculent, with entire edge, white to grey, reaching 25 mm in diam. after 10 days at 25 °C, forming umber conidiomata with cream conidial masses; colonies on SNA flat, spreading, with sparse aerial mycelium and undulate margin, white, reaching 25 mm in diam. after 20 days at 25 °C.

##### Additional material examined.

CHINA • Guangdong Province, Qingyuan City, Yangshan County, Guangdong Nanling Nature Reserve, on diseased leaves of *Castanopsis chunii*, 28 November 2019, Dan-ran Bian (culture CFCC 55908).

##### Distribution.

China, Guangdong Province.

##### Ecology.

Associated with leaf spot disease of *Castanopsis chunii*.

##### Notes.

Phylogenetically, *Diaporthe castanopsidicola* forms a highly supported sister clade to *D. linzhiensis* (Fig. 4). Although the mPTP analysis conservatively lumped these two taxa (along with *D. virgiliae*, *D. alnicola*, and *D. heterophyllae*), the PHI test (*p* = 1.0) confirmed no significant recombination between them (Fig. 5A), supporting their divergence. Morphologically, *D. castanopsidicola* can be readily distinguished from *D. linzhiensis* by its larger conidiomata (800–2000 μm in *D. castanopsidicola* vs. 300–500 μm in *D. linzhiensis*) (Li et al. 2025). Furthermore, comparisons of nucleotide sequences revealed significant differences between the two species: ITS (6/460 bp, 1.3%), *cal* (4/417 bp, 1.0%), *his3* (9/428 bp, 2.1%), *tef1* (4/335 bp, 1.2%), and *tub2* (6/428 bp, 1.4%). Based on the distinct morphology, significant genetic distance, and genealogical exclusivity, we introduce *D. castanopsidicola* as a new species.

#### Diaporthe changjiangensis

Taxon classificationAnimaliaDiaporthalesDiaporthaceae

Ning Jiang sp. nov.

F6951229-C2A9-5918-9C45-DA431CA5A6C0

848340

Fig. 9

##### Etymology.

Referring to Changjiang County, Hainan Province, where the type specimen was collected.

##### Diagnosis.

Distinct from its phylogenetically related species *D. yunnanensis* by wider alpha conidia.

##### Typus.

CHINA • Hainan Province, Changjiang Li Autonomous County, Bawangling National Forest Park, on diseased leaves of *Cyclobalanopsis kerrii*, 30 March 2019, Yong Li (holotype CAF800077; ex-holotype culture CFCC 54311).

##### Description.

***Conidiomata*** pycnidial, aggregated, erumpent, pulvinate, subglobose, brown, 150–450 μm diam. ***Conidiophores*** indistinct, usually reduced to conidiogenous cells. ***Conidiogenous cells*** hyaline, smooth, cylindrical, phialidic, 9–46 × 2.5–5.5 μm. ***Alpha conidia*** aseptate, hyaline, smooth, biguttulate, cylindrical, straight, base truncate, (5–)5.5–6.5(–7.5) × 2.5–3 μm (n = 50), L/W = 1.8–2.8.

**Figure 9.**
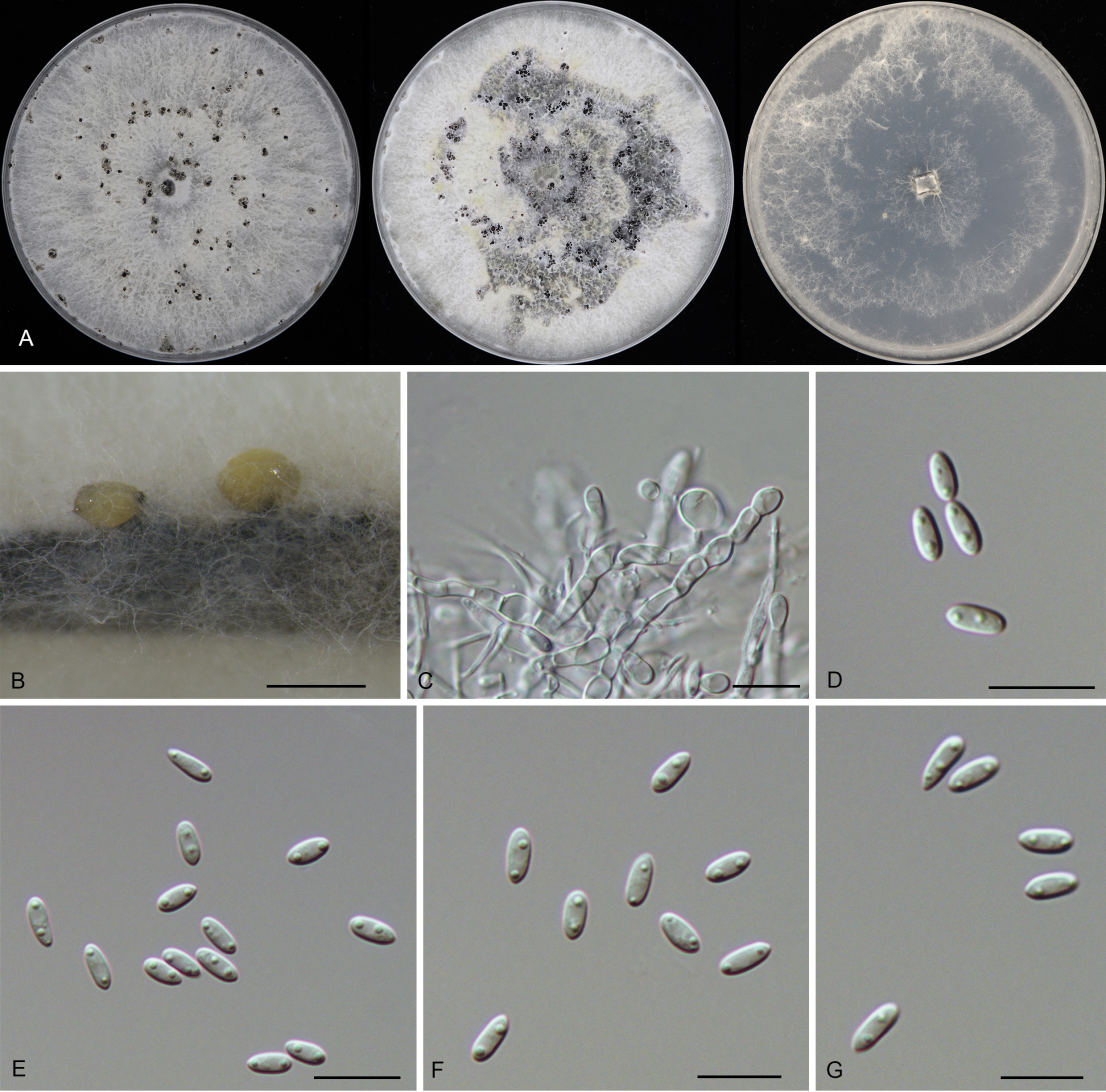
Morphology of *Diaporthe changjiangensis* (CFCC 54311). **A** Colonies on PDA, MPA and SNA after 20 d at 25 °C. **B** Conidiomata. **C, D** Conidiogenous cells. **D–G** Alpha conidia. Scale bars: 200 μm (**B**); 10 μm (**C–G**).

##### Culture characteristics.

Colonies on PDA flat, spreading, flocculent, with entire edge, white, reaching 25 mm in diam. after 10 days at 25 °C, forming brown conidiomata with luteous conidial masses; colonies on MPA flat, spreading, flocculent, with entire edge, white, forming dark brown irregular zone, reaching 25 mm in diam. after 10 days at 25 °C, forming brown conidiomata with luteous conidial masses; colonies on SNA flat, spreading, with sparse aerial mycelium and undulate margin, white, reaching 25 mm in diam. after 15 days at 25 °C, forming brown conidiomata with luteous conidial masses on the pine needles.

##### Additional material examined.

CHINA • Hainan Province, Changjiang Li Autonomous County, Bawangling National Forest Park, on diseased leaves of *Cyclobalanopsis kerrii*, 30 March 2019, Yong Li (culture CFCC 55917).

##### Distribution.

China, Hainan Province.

##### Ecology.

Associated with leaf spot disease of *Cyclobalanopsis kerrii*.

##### Notes.

In the phylogenetic analysis, *Diaporthe changjiangensis* forms a sister lineage to *D. yunnanensis* (Fig. 3). Although the PTP and mPTP analyses produced ambiguous species boundaries for this pair (Fig. 3), the PHI test (*p* = 1.0) strongly supported the lack of recombination between them (Fig. 5B). Morphologically, *D. changjiangensis* differs from *D. yunnanensis* (described from *Coffea* sp.) by having distinctly wider alpha conidia (2.5–3 μm vs. 1–2.5 μm) (Gao et al. 2017). Most notably, comparison of nucleotide sequences revealed remarkable genetic divergence between the two species, particularly in the ITS region (23/462 bp, 5.0%), as well as in *his3* (9/453 bp, 2.0%) and *tef1* (3/338 bp, 0.9%). Such a high degree of sequence variation in the ITS region provides evidence for the establishment of *D. changjiangensis* as a novel species.

#### Diaporthe cyclobalanopsidicola

Taxon classificationAnimaliaDiaporthalesDiaporthaceae

Ning Jiang sp. nov.

8AC20099-3ED5-5258-923B-B147C3CD1249

841316

Fig. 10

##### Etymology.

Named after the host genus *Cyclobalanopsis* and “-*cola*” = “inhabiting”.

##### Diagnosis.

Distinct from *D. aseana* by longer alpha conidia and from *D. xishuangbanica* by larger conidiomata.

##### Typus.

CHINA • Hainan Province, Changjiang Li Autonomous County, Bawangling National Forest Park, on diseased leaves of *Cyclobalanopsis blakei*, 30 March 2019, Yong Li (holotype CAF800029; ex-type culture CFCC 54462).

##### Description.

***Conidiomata*** pycnidial, aggregated or solitary, erumpent, subglobose, brown, 500–1300 μm diam. ***Conidiophores*** indistinct, usually reduced to conidiogenous cells. ***Conidiogenous cells*** hyaline, smooth, cylindrical, phialidic, 11.5–57 × 1.5–2.5 μm. ***Alpha conidia*** aseptate, hyaline, smooth, guttulate, fusoid, straight or slightly curved, base truncate, (8–)8.5–12.5(–15) × (2–)2.5–3(–3.5) μm (n = 50), L/W = 2.5–5. ***Beta conidia*** aseptate, hyaline, smooth, guttulate, filiform, straight or curved, (17.5–)20.5–29.5(–32) × 1.5–2 μm (n = 50), L/W = 10.3–19.9.

**Figure 10.**
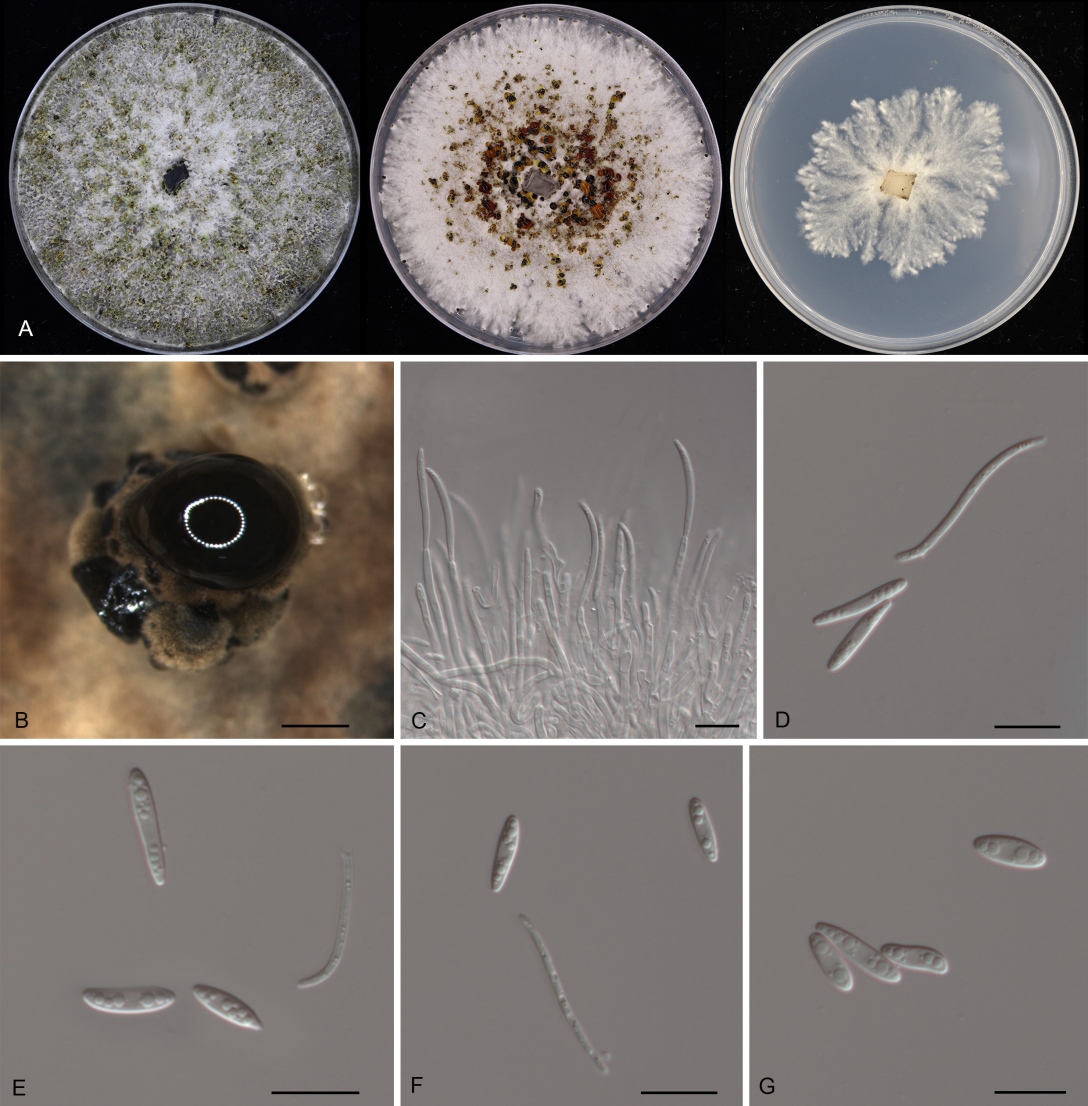
Morphology of *Diaporthe cyclobalanopsidicola* (CFCC 54462). **A** Colonies on PDA, MPA and SNA after 20 d at 25 °C. **B** Conidiomata. **C** Conidiogenous cells giving rise to conidia. **D–G** Alpha and beta conidia. Scale bars: 400 μm (**B**); 10 μm (**C–G**).

##### Culture characters.

Colonies on PDA flat, spreading, flocculent, with entire edge, initially white, becoming olivaceous grey with age, reaching 25 mm in diam. after 7 days at 25 °C; colonies on MPA flat, spreading, flocculent, with entire edge, white to pale luteous, reaching 25 mm in diam. after 7 days at 25 °C, forming brown conidiomata with amber conidial masses; colonies on SNA flat, feathery, with sparse aerial mycelium and undulate margin, white.

##### Additional material examined.

CHINA • Hainan Province, Changjiang Li Autonomous County, Bawangling National Forest Park, on diseased leaves of *Cyclobalanopsis blakei*, 28 March 2019, Yong Li (culture CFCC 55903).

##### Distribution.

China, Hainan Province.

##### Ecology.

Associated with leaf spot disease of *Cyclobalanopsis blakei*.

##### Notes.

Phylogenetically, *Diaporthe cyclobalanopsidicola* resides in a subclade with *D. aseana* and *D. xishuangbanica* (Fig. 1). Although the mPTP analysis conservatively recognized this group as a single species, the PHI test (*p* = 0.447) detected no significant recombination (Fig. 5D), supporting their status as independent lineages. Morphologically, *D. cyclobalanopsidicola* is easily distinguished from *D. aseana* by its longer alpha conidia (8.5–12.5 × 2.5–3 μm in *D. cyclobalanopsidicola* vs. 6–9 × 2–3 μm in *D. aseana*) and from *D. xishuangbanica* by markedly larger conidiomata (500–1300 μm in *D. cyclobalanopsidicola* vs. 180–310 μm in *D. xishuangbanica*) (Hyde et al. 2016; Gao et al. 2017). Moreover, pairwise sequence alignment revealed substantial nucleotide divergence. *Diaporthe cyclobalanopsidicola* differs from *D. aseana* by 17 bp in ITS, 24 bp in *cal*, 19 bp in *his3*, 13 bp in *tef1*, and 38 bp in *tub2*. Similarly, it differs from *D. xishuangbanica* by 11 bp in ITS, 31 bp in *his3*, 31 bp in *tef1*, and 33 bp in *tub2*. These significant genetic distances provide robust evidence to justify *D. cyclobalanopsidicola* as a distinct species.

#### Diaporthe liangxii

Taxon classificationAnimaliaDiaporthalesDiaporthaceae

Ning Jiang sp. nov.

0AEB6BF0-E93C-503D-8615-B8219FD4470C

841318

Fig. 11

##### Etymology.

In honor of Prof. Dr. Liang Xi, in recognition of his distinguished contributions to forestry research in China.

##### Diagnosis.

Distinct from its phylogenetically related species of *D. zhaoqingensis* by host plant.

##### Typus.

CHINA • Hainan Province, Changjiang Li Autonomous County, Bawangling National Forest Park, on diseased leaves of *Lithocarpus naiadarum*, 30 March 2019, Yong Li (holotype CAF800078; ex-holotype culture CFCC 54324).

##### Description.

***Conidiomata*** pycnidial, aggregated, erumpent, pulvinate, subglobose, dark brown, 350–1000 μm diam. ***Conidiophores*** indistinct, usually reduced to conidiogenous cells. ***Conidiogenous cells*** hyaline, smooth, filiform, phialidic, 9.5–22.5 × 2–3.5 μm. ***Beta conidia*** aseptate, hyaline, smooth, guttulate or not, filiform, straight or hamate, base truncate, (13.5–)20.5–30(–31) × (1–)1.5–2 μm (n = 50), L/W = 14.1–23.2.

**Figure 11.**
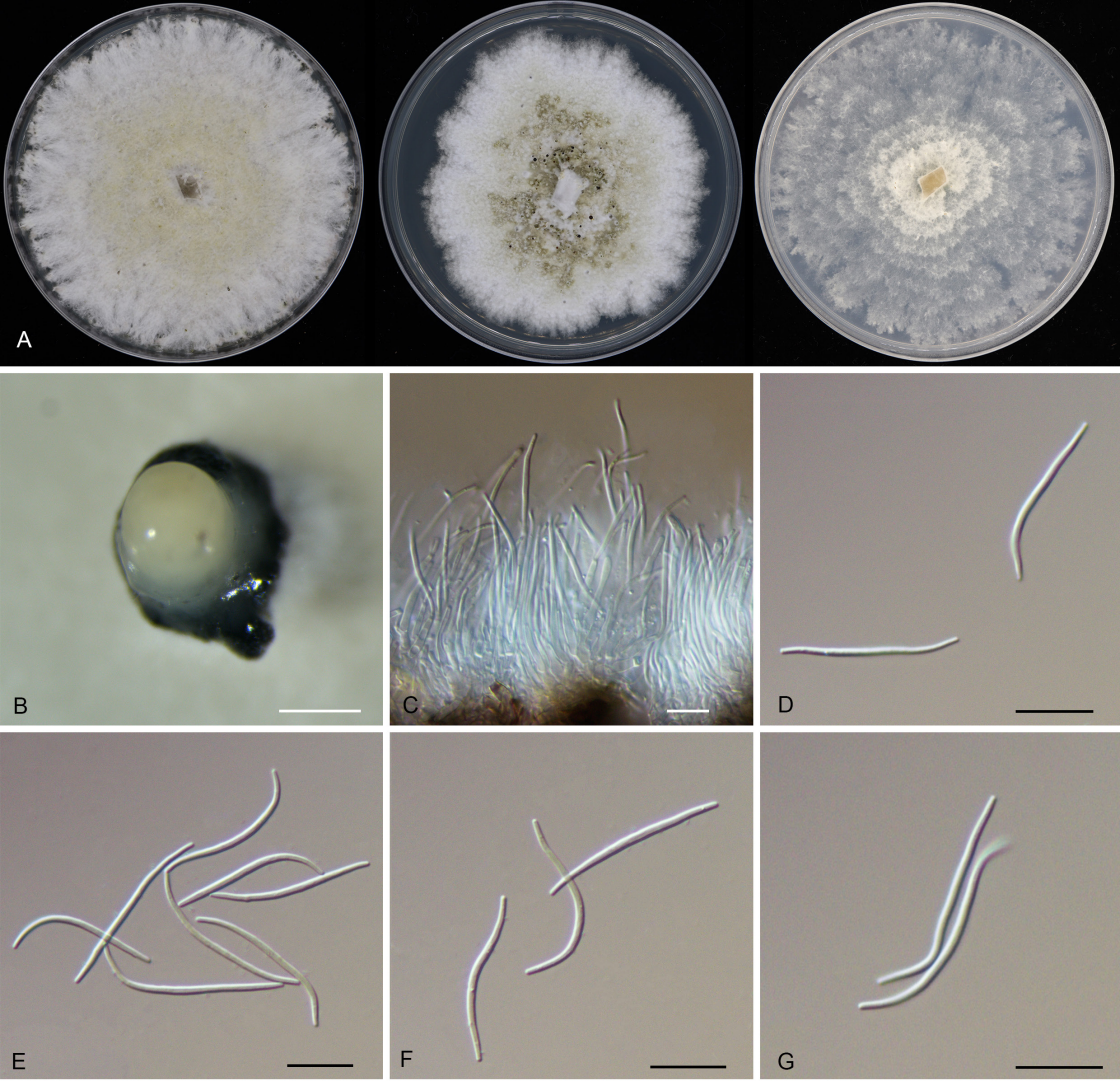
Morphology of *Diaporthe liangxii* (CFCC 54324). **A** Colonies on PDA, MPA and SNA after 20 d at 25 °C. **B** Conidioma. **C** Conidiogenous cells giving rise to conidia. **D–G** Beta conidia. Scale bars: **B** = 200 μm; **C–G** = 10 μm.

##### Culture characters.

Colonies on PDA flat, spreading, flocculent, with entire edge, white to buff, reaching 25 mm in diam. after 10 days at 25 °C; colonies on MPA flat, spreading, flocculent, with undulate edge, initially white, forming grey zones in the middle and producing dark brown conidiomata with cream conidial masses, reaching 25 mm in diam. after 10 days at 25 °C; colonies on SNA flat, spreading, with sparse aerial mycelium and undulate margin, white, forming concentric zones with age, reaching 25 mm in diam. after 15 days at 25 °C.

##### Additional material examined.

CHINA • Hainan Province, Changjiang Li Autonomous County, Bawangling National Forest Park, on diseased leaves of *Lithocarpus naiadarum*, 30 March 2019, Yong Li (culture CFCC 55901).

##### Distribution.

China, Hainan Province.

##### Ecology.

Associated with leaf spot disease of *Lithocarpus naiadarum*.

##### Notes.

Phylogenetically, *Diaporthe liangxii* clusters with *D. zhaoqingensis* (Fig. 1). Due to their close relationship, the mPTP analysis lumped them into a single entity; however, the PHI test (p = 0.958) revealed no significant recombination (Fig. 5E), supporting their separation as distinct lineages. Morphologically, *D. liangxii* (producing only beta conidia in this study) shares similar conidial dimensions with *D. zhaoqingensis* (Luo et al. 2022). Nevertheless, they can be distinguished by host association (*Lithocarpus naiadarum* vs. *Morinda officinalis*). More importantly, the two species exhibit remarkable genetic divergence. *Diaporthe liangxii* differs from *D. zhaoqingensis* by 12 bp in ITS, 45 bp in *cal*, 3 bp in *his3*, and 34 bp in *tub2*. The substantial sequence variations in protein-coding genes (*cal* and *tub2*) strongly justify the recognition of *D. liangxii* as a novel species despite their morphological resemblance.

#### Diaporthe lithocarpicola

Taxon classificationAnimaliaDiaporthalesDiaporthaceae

Ning Jiang sp. nov.

CB5B039E-33E5-5113-BB6C-C14828CC4B58

841319

Fig. 12

##### Etymology.

Named after the host genus *Lithocarpus* and “-*cola*” = “inhabiting”.

##### Diagnosis.

Distinct from its phylogenetically related species *D. sennicola* by wider conidiogenous cells.

##### Typus.

CHINA • Hainan Province, Changjiang Li Autonomous County, Bawangling National Forest Park, on diseased leaves of *Lithocarpus naiadarum*, 28 March 2019, Yong Li (holotype CAF800079; ex-holotype culture CFCC 54297).

##### Description.

***Conidiomata*** pycnidial, aggregated or solitary, erumpent, subglobose, amber, 400–950 μm diam. ***Conidiophores*** indistinct, usually reduced to conidiogenous cells. ***Conidiogenous cells*** hyaline, smooth, cylindrical to ampulliform, attenuate towards the apex, phialidic, 4.5–13.5 × 1.5–2.5 μm. ***Beta conidia*** aseptate, hyaline, smooth, guttulate or not, filiform, curved, (24.5–)28.5–38(–43.5) × 1.5–2 μm (n = 50), L/W = 15.4–27.9.

**Figure 12.**
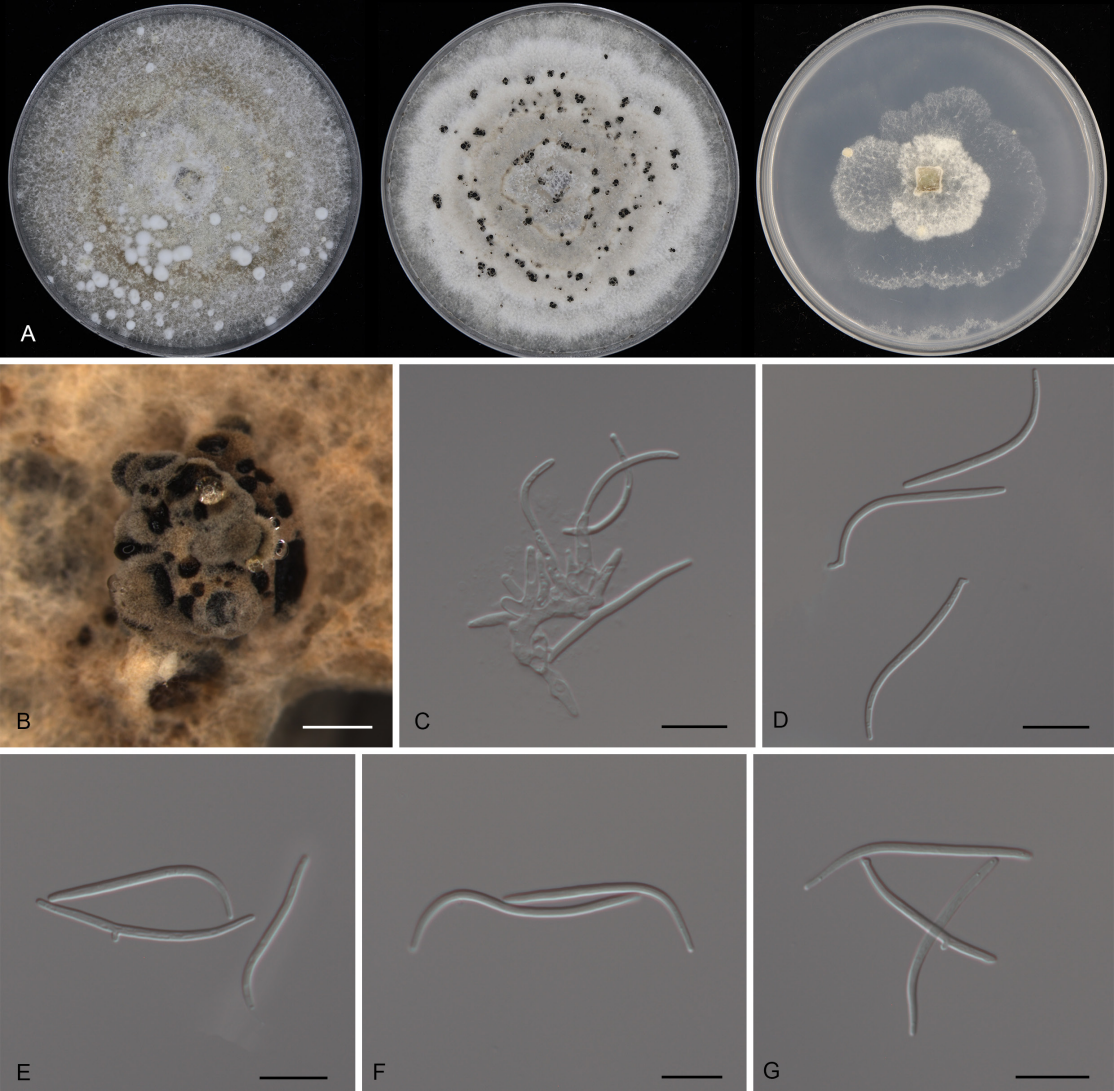
Morphology of *Diaporthe lithocarpicola* (CFCC 54297). **A** Colonies on PDA, MPA and SNA after 20 d at 25 °C. **B** Conidiomata. **C** Conidiogenous cells giving rise to conidia. **D–G** Beta conidia. Scale bars: 400 μm (**B**); 10 μm (**C–G**).

##### Culture characters.

Colonies on PDA flat, spreading, flocculent, with entire edge, initially white, becoming olivaceous grey with age, reaching 25 mm in diam. after 10 days at 25 °C; colonies on MPA flat, spreading, flocculent, with entire edge, white to amber, reaching 25 mm in diam. after 10 days at 25 °C, forming amber conidiomata with brown conidial masses; colonies on SNA flat, with sparse aerial mycelium and undulate margin, white, reaching 25 mm in diam. after 20 days at 25 °C.

##### Additional material examined.

CHINA • Changjiang Li Autonomous County, Bawangling National Forest Park, on diseased leaves of *Lithocarpus naiadarum*, 30 March 2019, Yong Li (culture CFCC 55913).

##### Ecology.

Associated with leaf spot disease of *Lithocarpus naiadarum*.

##### Notes.

Phylogenetically, *Diaporthe lithocarpicola* is closely related to *D. sennicola* (Fig. 2). Both PTP and mPTP analyses produced ambiguous species boundaries between *D. lithocarpicola* and *D. sennicola*. However, the PHI test (*p* = 1.0) provided strong evidence for the lack of recent recombination (Fig. 5C), supporting their genealogical independence. Morphologically, *D. lithocarpicola* differs significantly from *D. sennicola* (on *Senna bicapsularis*) by having much wider conidiogenous cells (1.5–2.5 μm vs. 0.9–1.4 μm) (Yang et al. 2017). Genetic comparison further justifies their separation, despite high similarity in the ITS region (1/462 bp difference). *Diaporthe lithocarpicola* exhibits distinct variations in protein-coding genes, differing from *D. sennicola* by 13 bp in *cal*, 14 bp in *tef1*, 5 bp in *tub2*, and 2 bp in *his3*.

#### Diaporthe siamensis

Taxon classificationAnimaliaDiaporthalesDiaporthaceae

Udayanga, Xing Z. Liu & K.D. Hyde, Cryptog. Mycol. 33(3): 298 (2012)

CAAC3F4F-2F58-5446-B5D1-5DFF3C6C1B66

Fig. 13

##### Description.

See Udayanga et al. (2012).

##### Distribution.

China, Thailand.

##### Ecology.

Associated with leaf spot disease of *Cyclobalanopsis kerrii*, *Dasymaschalon* sp., and *Lithocarpus levis*, and fruit rot disease of *Citrus sinensis*.

##### Materials examined.

CHINA • Hainan Province, Changjiang Li Autonomous County, Bawangling National Forest Park, on diseased leaves of *Cyclobalanopsis kerrii*, 30 March 2019, Yong Li (cultures CFCC 54817, CFCC 55916). Hainan Province, Changjiang Li Autonomous County, Bawangling National Forest Park, on diseased leaves of *Lithocarpus levis*, 30 March 2019, Yong Li (culture CFCC 54295).

**Figure 13.**
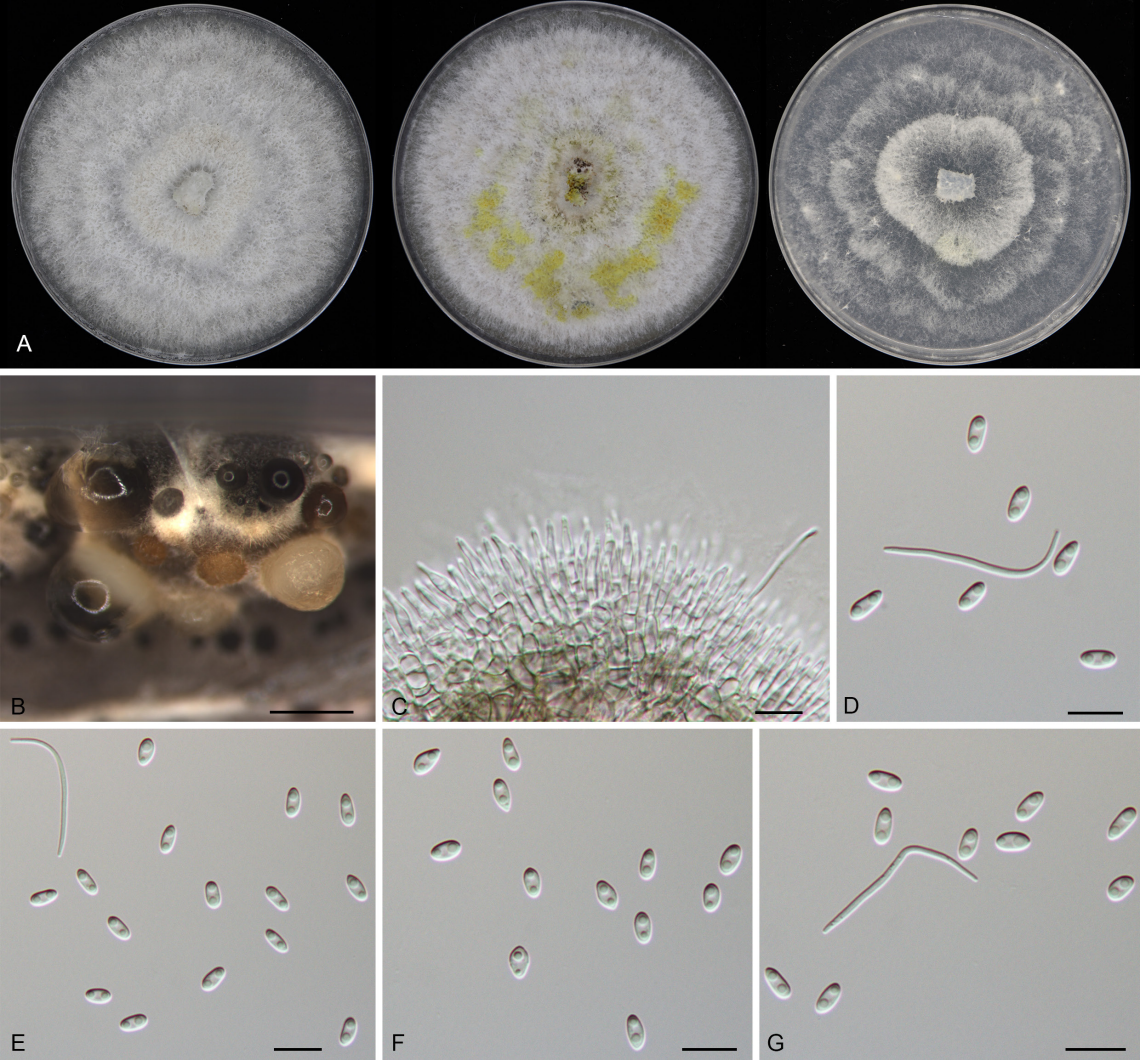
Morphology of *Diaporthe siamensis* (CFCC 54295). **A** Colonies on PDA, MPA and SNA after 20 d at 25 °C. **B** Conidiomata. **C** Conidiogenous cells. **D–G** Alpha and beta conidia. Scale bars: 300 μm (**B**); 10 μm (**C–G**).

##### Notes.

*Diaporthe siamensis* was originally described from *Dasymaschalon* sp. (Annonaceae) in Thailand (Udayanga et al. 2012). In the current study, three isolates recovered from *Cyclobalanopsis kerrii* and *Lithocarpus levis* clustered with the ex-type strain of *D. siamensis* (MFLUCC 10-0573a) with maximum statistical support (ML/BI = 100/1) (Fig. 3). These findings constitute new host records for *D. siamensis* on *Cyclobalanopsis* and *Lithocarpus* species, broadening the known host range of this pathogen to the family *Fagaceae*.

## Discussion

*Diaporthe* is widely recognized as one of the most speciose and ecologically diverse genera within the *Ascomycota*. While extensive surveys have been conducted on economic crops (e.g., *Citrus*, *Helianthus*, *Vitis*) and ornamental plants (Gomzhina and Gannibal 2022; Wan et al. 2023), the diversity of *Diaporthe* associated with forest ecosystems, particularly in subtropical regions, remains significantly underestimated. *Fagaceae* species (e.g., *Castanopsis*, *Lithocarpus*, *Cyclobalanopsis*) are dominant canopy trees in the evergreen broad-leaved forests of southern China. Our study revealed a remarkable diversity of *Diaporthe* species inhabiting foliar lesions of these trees. From a relatively localized survey, we identified eight distinct lineages of *Diaporthe*, five of which are described here as new to science. This high rate of novelty (62.5%) suggests that *Fagaceae* forests serve as a substantial reservoir for *Diaporthe* diversity. Our findings corroborate the hypothesis that the “hyper-diversity” of *Diaporthe* is largely driven by its association with diverse woody hosts in natural ecosystems, warranting broader sampling efforts in forestry pathology.

Accurate species delimitation in *Diaporthe* has long been impeded by morphological plasticity and the lack of informative barcodes. In recent years, coalescence-based methods (GMYC, PTP/mPTP) have become standard tools for establishing species boundaries (Hilário et al. 2020, 2021a, 2021b; Dissanayake et al. 2024; Pereira et al. 2023; Pereira and Phillips 2024). However, our study highlights the limitations of relying solely on automated delimitation algorithms. In the *D. virgiliae* species complex, the mPTP model conservatively lumped *D. castanopsidicola* with clearly distinct species such as *D. virgiliae* and *D. alnicola* (Fig. 4). Similar incongruences were observed in the *D. arecae*, *D. gardeniae*, and *D. siamensis* complexes. While mPTP is generally considered robust, it can be prone to “over-lumping” when analyzing datasets with uneven sampling or recent rapid radiations (Kapli et al. 2017). To resolve these conflicts, we adopted a rigorous integrative taxonomic approach. We prioritized GCPSR based on well-supported clades in multi-locus phylogeny (ML/BI), supported by pairwise homoplasy index (PHI) tests which confirmed the absence of recent recombination (p > 0.05). Furthermore, significant nucleotide divergence in protein-coding genes (particularly *tub2* and *tef1*) and distinct morphological traits (e.g., conidiomata size in *D. castanopsidicola* vs. *D. linzhiensis*) provided irrefutable evidence for the validity of the five new species. This study serves as a case study demonstrating that algorithmic delimitation results should be interpreted with caution and verified against biological and genetic distance data.

Cryptic speciation is a prevalent phenomenon in *Diaporthe* (Yang et al. 2018; Zhu et al. 2024). Traditional identification based on host association or ITS sequences often fails to distinguish closely related taxa (Yang et al. 2018; Dissanayake et al. 2024; Zhu et al. 2024). For instance, in our study, *D. lithocarpicola* and *D. sennicola* share high similarity in the ITS region (only 1 bp difference), which might lead to misidentification if analyzing ITS alone. However, the inclusion of *cal* and *tef1* revealed substantial divergence (13–14 bp), disentangling these cryptic lineages. Similarly, *D. liangxii* and *D. zhaoqingensis* are morphologically indistinguishable in their anamorphs but are genetically distinct in *cal* (45 bp) and *tub2* (34 bp). Conversely, *D. changjiangensis* exhibited an unexpectedly high variation in ITS (23 bp) compared to its sister species *D. yunnanensis*. These findings reinforce the necessity of using a five-locus concatened dataset (ITS, *cal*, *his3*, *tef1*, *tub2*) for reliable species resolution in *Diaporthe*, as proposed by recent taxonomic frameworks (Dissanayake et al. 2024).

The host range of *Diaporthe* species varies from strictly host-specific to broadly generalist (Mostert et al. 2001; Santos and Phillips 2009; Dissanayake et al. 2024). In this study, the five novel species (*D. castanopsidicola*, *D. changjiangensis*, *D. cyclobalanopsidicola*, *D. liangxii*, and *D. lithocarpicola*) were isolated exclusively from specific *Fagaceae* hosts (*Castanopsis*, *Cyclobalanopsis*, and *Lithocarpus*), suggesting potential host preference. However, given the limited sampling, it is premature to conclude strict host specificity. In contrast, we also recovered *D. apiculata* and *D. siamensis* from *Fagaceae* leaf spots. *Diaporthe apiculata* has been previously reported on *Camellia (Theaceae)* and *Rhus (Anacardiaceae)*, and *D. siamensis* on *Citrus (Rutaceae)* and *Dasymaschalon (Annonaceae)*. The identification of these known species on *Fagaceae* represent new host records and suggest they are generalist pathogens capable of cross-infection between forest trees and economic crops. This “host-jumping” potential poses a challenge for disease management in mixed-plantation forestry and highlights the need for further pathogenicity tests to evaluate their virulence across different host families.

## Supplementary Material

XML Treatment for Diaporthe apiculata

XML Treatment for Diaporthe arecae

XML Treatment for Diaporthe castanopsidicola

XML Treatment for Diaporthe changjiangensis

XML Treatment for Diaporthe cyclobalanopsidicola

XML Treatment for Diaporthe liangxii

XML Treatment for Diaporthe lithocarpicola

XML Treatment for Diaporthe siamensis

## Additional information

### Artificial Intelligence (AI) use

The authors accept full responsibility for the content of the manuscript, including the disclosure of any use of AI.

No AI tools were used in the preparation of this manuscript.

### Adherence to national and international regulations

All the fungal strains used in this study have been legally obtained, respecting the Convention on Biological Diversity (Rio Convention).

### Author ORCIDs

X. Zhong https://orcid.org/0009-0005-6817-2006

H. Voglmayr https://orcid.org/0000-0001-7666-993X

H. Xue https://orcid.org/0000-0003-0414-6237

Y. Li https://orcid.org/0000-0002-4406-1329

N. Jiang https://orcid.org/0000-0002-9656-8500

### Data availability

All of the data that support the findings of this study are available in the main text or Supplementary Information.

## Supplementary materials

Supplementary material 1 Phylogram of *Diaporthe* resulting from a maximum likelihood analysis based on the *ITS*, *cal*, *his3*, *tef1* and *tub2* gene loci Data type pdf https://binary.pensoft.net/file/1646178This dataset is made available under the Open Database License (http://opendatacommons.org/licenses/odbl/1.0/). The Open Database License (ODbL) is a license agreement intended to allow users to freely share, modify, and use this Dataset while maintaining this same freedom for others, provided that the original source and author(s) are credited. Ning Jiang

Supplementary material 2 Single-gene phylogenies Data type docx https://binary.pensoft.net/file/1646179This dataset is made available under the Open Database License (http://opendatacommons.org/licenses/odbl/1.0/). The Open Database License (ODbL) is a license agreement intended to allow users to freely share, modify, and use this Dataset while maintaining this same freedom for others, provided that the original source and author(s) are credited. Ning Jiang

Supplementary material 3 Alignments of arecae species complex Data type fas https://binary.pensoft.net/file/1646180This dataset is made available under the Open Database License (http://opendatacommons.org/licenses/odbl/1.0/). The Open Database License (ODbL) is a license agreement intended to allow users to freely share, modify, and use this Dataset while maintaining this same freedom for others, provided that the original source and author(s) are credited. Ning Jiang

Supplementary material 4 Alignments of gardeniae species complex Data type fas https://binary.pensoft.net/file/1646181This dataset is made available under the Open Database License (http://opendatacommons.org/licenses/odbl/1.0/). The Open Database License (ODbL) is a license agreement intended to allow users to freely share, modify, and use this Dataset while maintaining this same freedom for others, provided that the original source and author(s) are credited. Ning Jiang

Supplementary material 5 Alignments of siamensis species complex Data type fas https://binary.pensoft.net/file/1646182This dataset is made available under the Open Database License (http://opendatacommons.org/licenses/odbl/1.0/). The Open Database License (ODbL) is a license agreement intended to allow users to freely share, modify, and use this Dataset while maintaining this same freedom for others, provided that the original source and author(s) are credited. Ning Jiang

Supplementary material 6 Alignments of virgiliae species complex Data type fas https://binary.pensoft.net/file/1646183This dataset is made available under the Open Database License (http://opendatacommons.org/licenses/odbl/1.0/). The Open Database License (ODbL) is a license agreement intended to allow users to freely share, modify, and use this Dataset while maintaining this same freedom for others, provided that the original source and author(s) are credited. Ning Jiang
