# Supplementary figures and images for: Unveiling cryptic diversity of Diaporthe associated with leaf spots of Fagaceae in China using an integrative taxonomic approach
Source: IMA Fungus. 2026 May 15;17:e186438. doi: 10.3897/imafungus.17.186438 (PMC13197814; doi:10.3897/imafungus.17.186438)

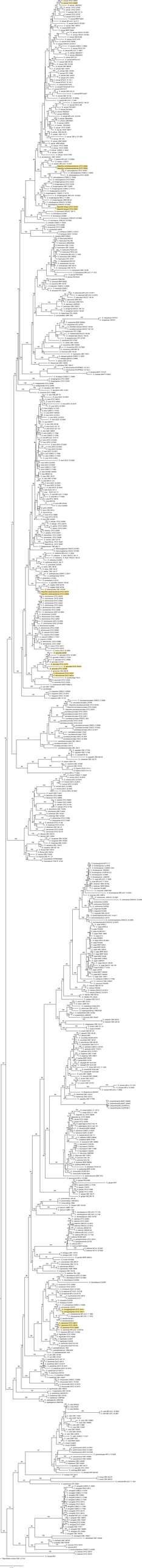

Supplement: Supplementary material 1 — Phylogram of Diaporthe resulting from a maximum likelihood analysis based on the ITS, cal, his3, tef1 and tub2 gene loci [file imafungus-17-e186438-s001.pdf]
