# Supplementary material for: Unveiling cryptic diversity of Diaporthe associated with leaf spots of Fagaceae in China using an integrative taxonomic approach
Source: IMA Fungus. 2026 May 15;17:e186438. doi: 10.3897/imafungus.17.186438 (PMC13197814; doi:10.3897/imafungus.17.186438)
Supplement: Supplementary material 2 — Single-gene phylogenies [file imafungus-17-e186438-s002.docx]

**Single-gene phylogenies**


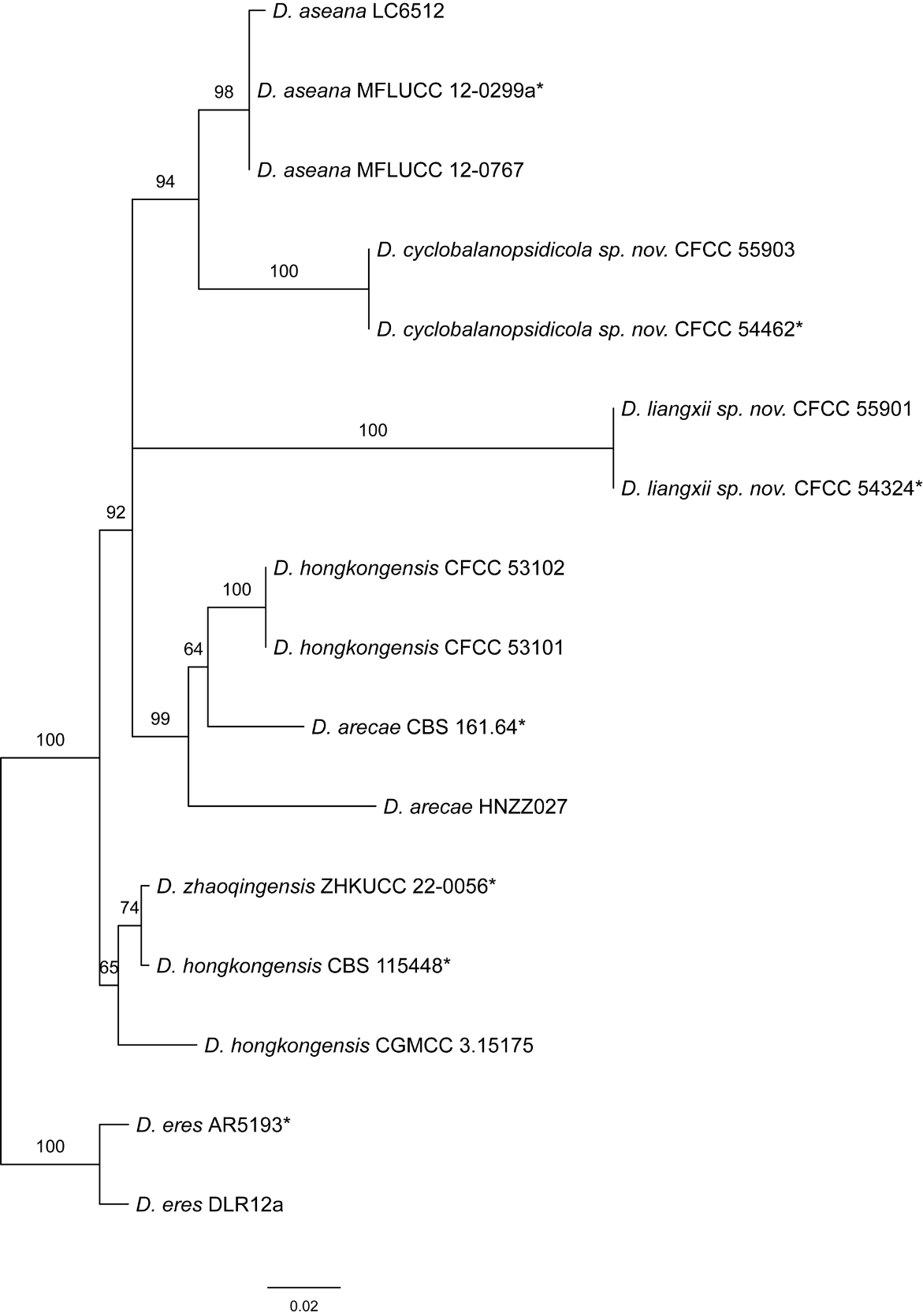


**Fig.** **S1** Phylogram of *Diaporthe arecae* and related species resulting from a maximum likelihood analysis based on the *cal* gene. Numbers above the branches indicate ML bootstrap values. Ex-type strains are marked with *. The scale bar represents the expected number of nucleotide substitutions per site. The tree is rooted with *D. eres* (AR5193 and DLR12a).


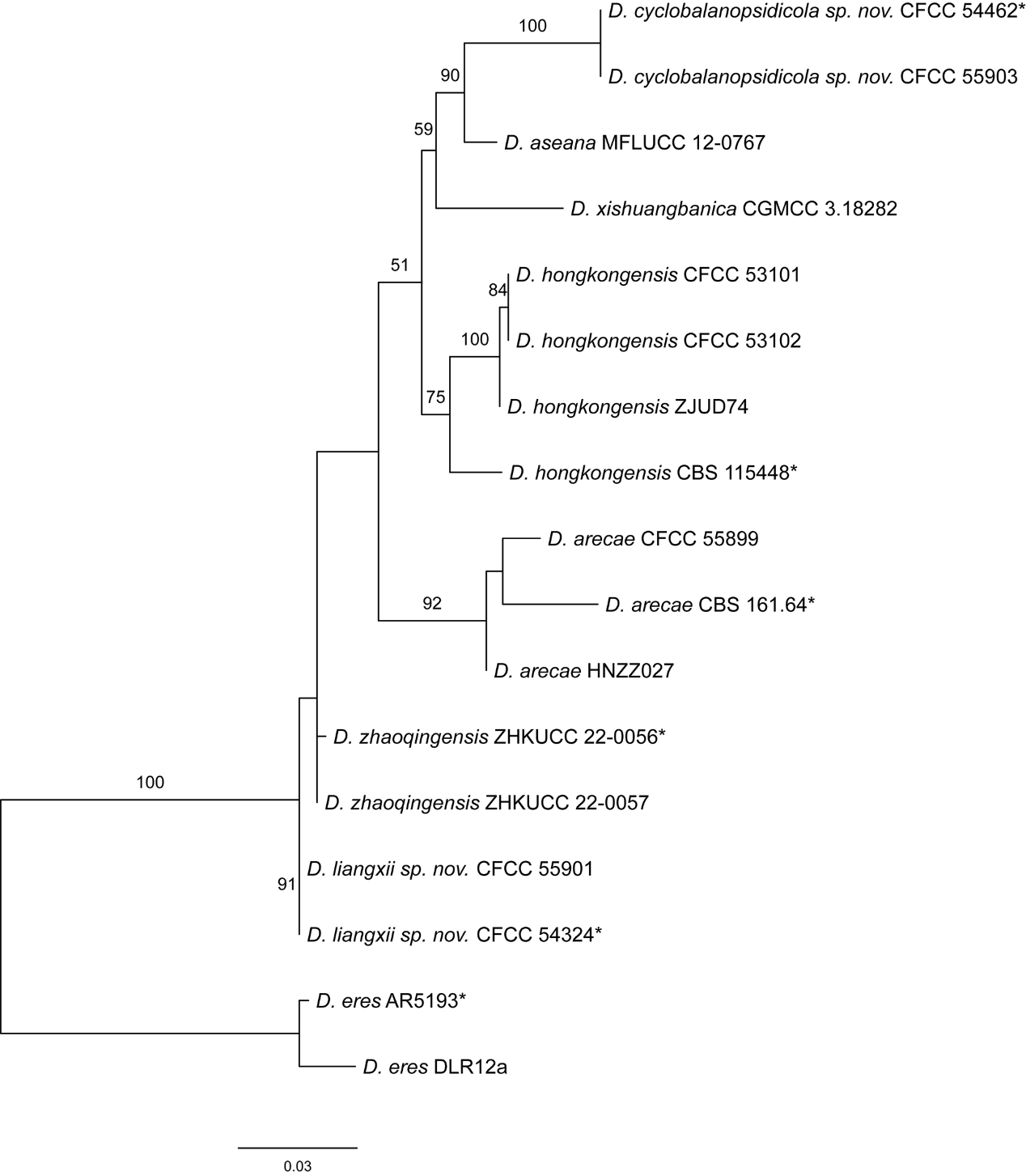


**Fig.** **S2** Phylogram of *Diaporthe arecae* and related species resulting from a maximum likelihood analysis based on the *his3* gene. Numbers above the branches indicate ML bootstrap values. Ex-type strains are marked with *. The scale bar represents the expected number of nucleotide substitutions per site. The tree is rooted with *D. eres* (AR5193 and DLR12a).


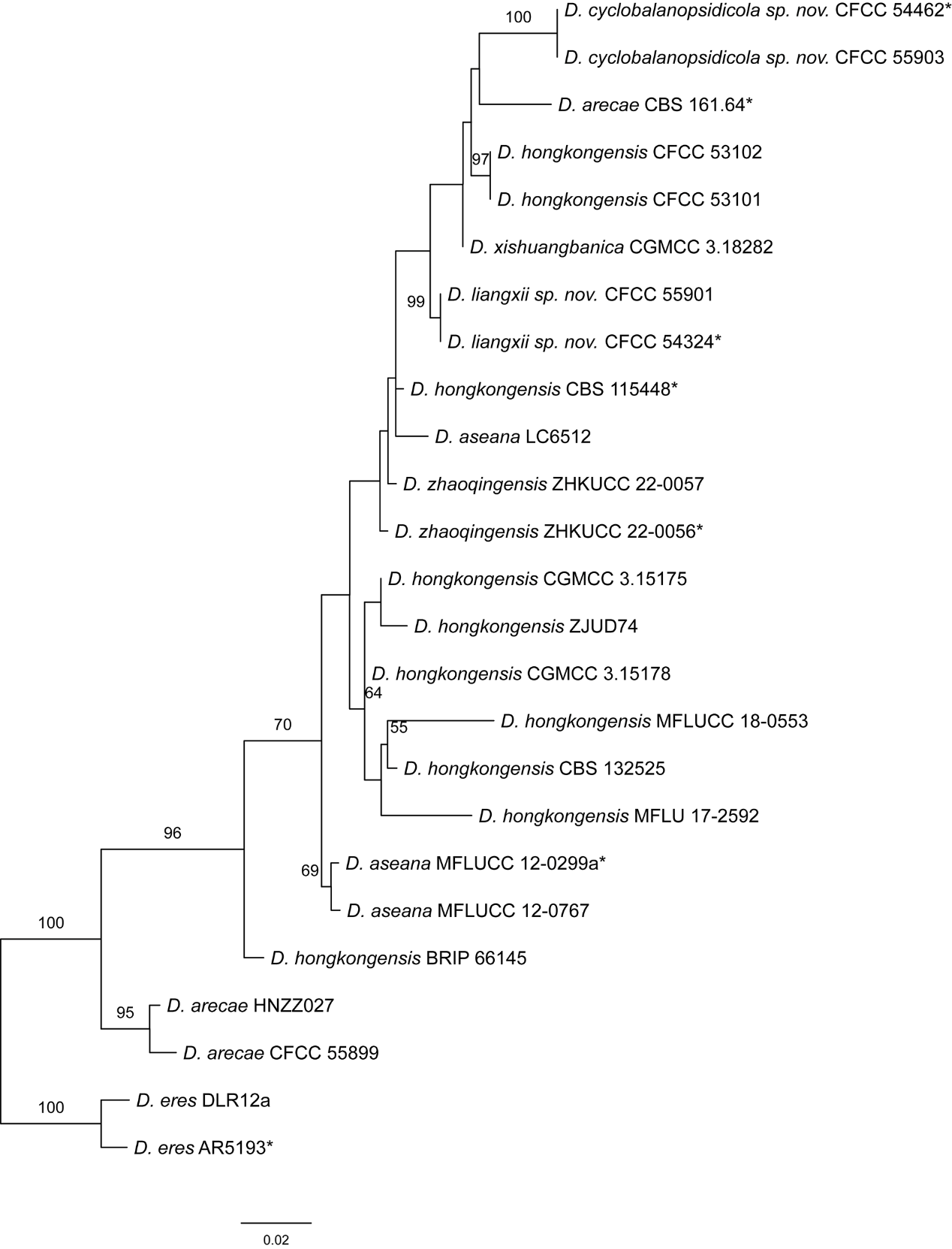


**Fig.** **S3** Phylogram of *Diaporthe arecae* and related species resulting from a maximum likelihood analysis based on the ITS sequence. Numbers above the branches indicate ML bootstrap values. Ex-type strains are marked with *. The scale bar represents the expected number of nucleotide substitutions per site. The tree is rooted with *D. eres* (AR5193 and DLR12a).


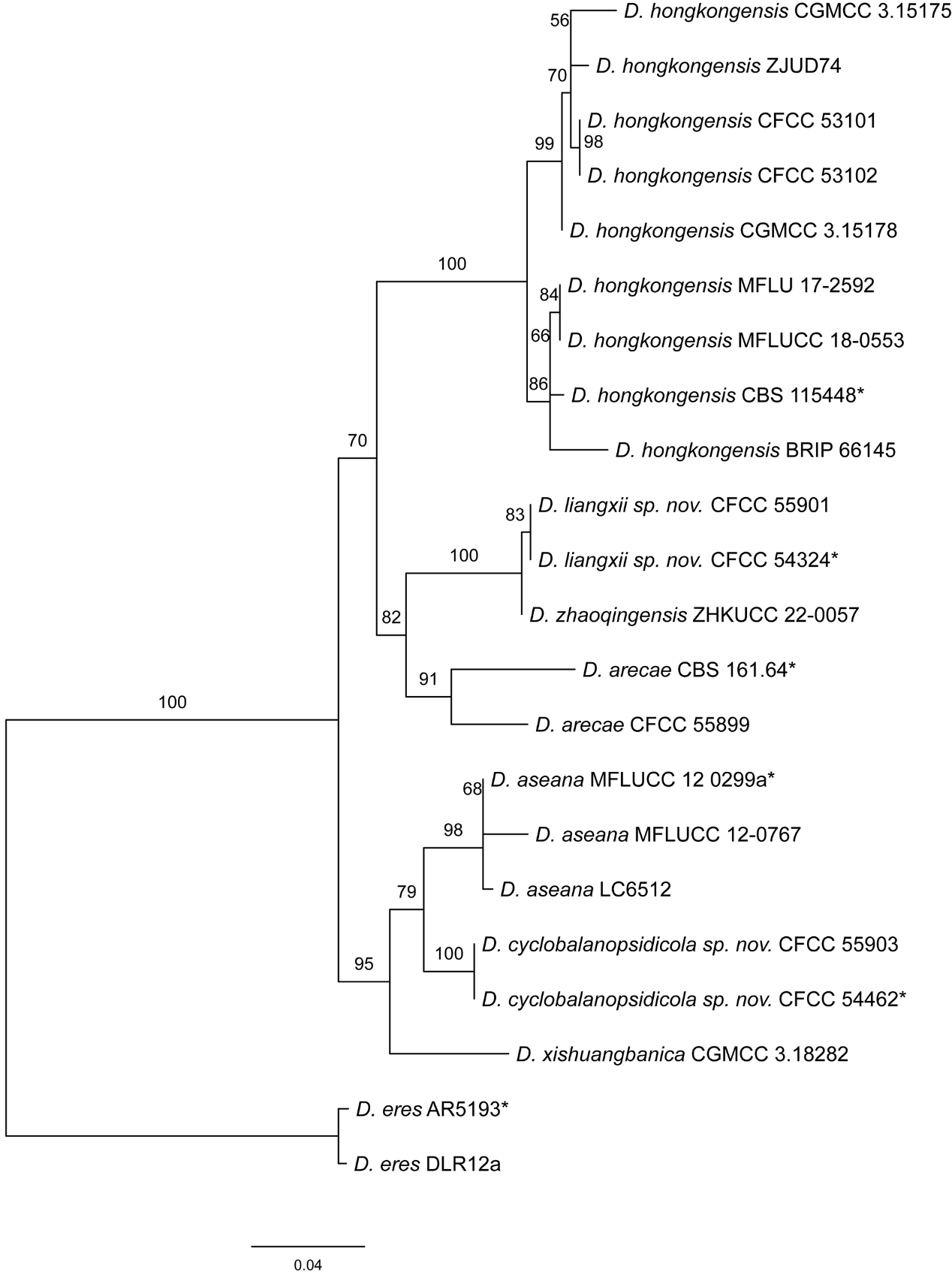


**Fig.** **S4** Phylogram of *Diaporthe arecae* and related species resulting from a maximum likelihood analysis based on the *tef1* gene. Numbers above the branches indicate ML bootstrap values. Ex-type strains are marked with *. The scale bar represents the expected number of nucleotide substitutions per site. The tree is rooted with *D. eres* (AR5193 and DLR12a).


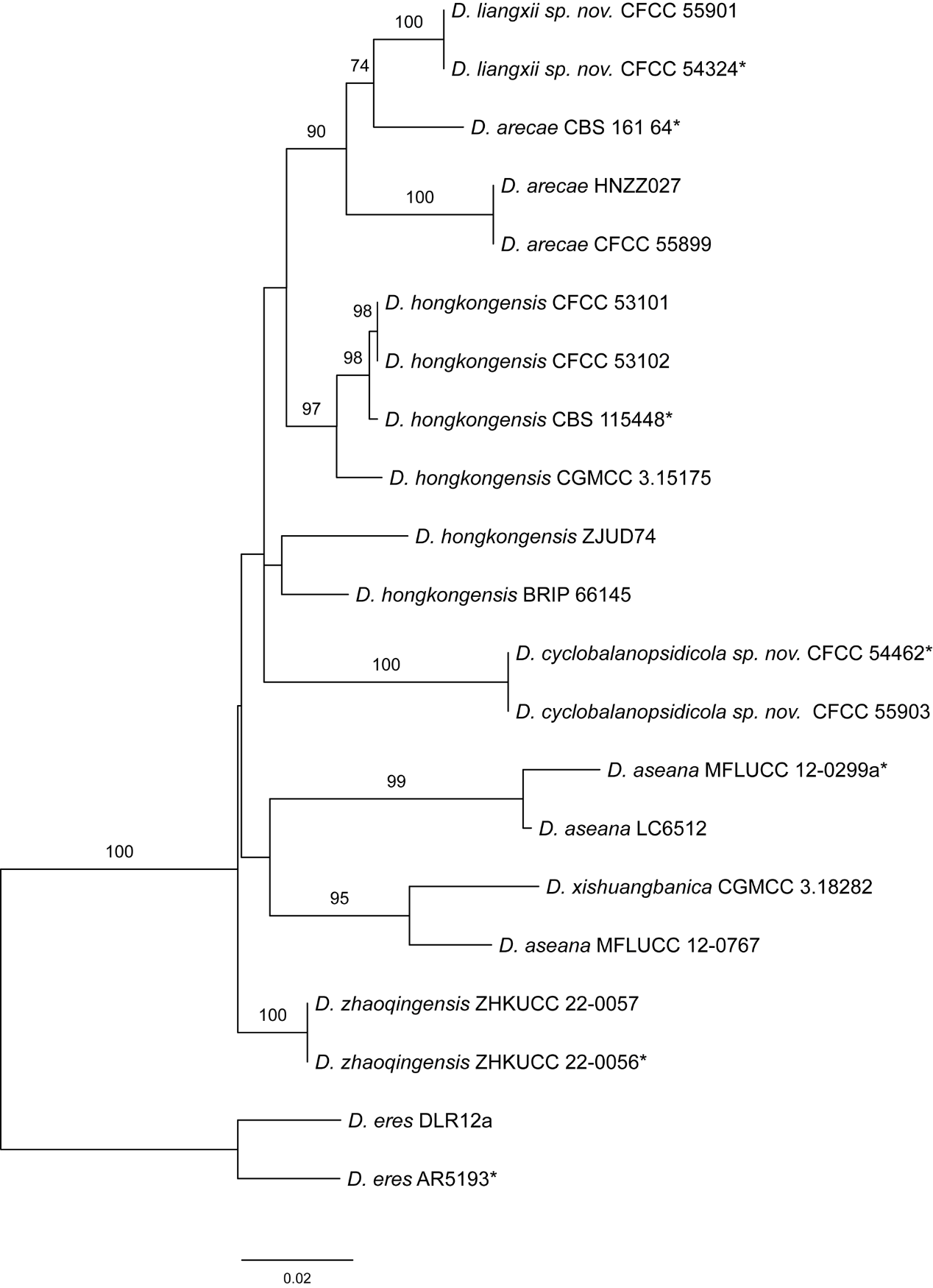


**Fig.** **S5** Phylogram of *Diaporthe arecae* and related species resulting from a maximum likelihood analysis based on the *tub2* gene. Numbers above the branches indicate ML bootstrap values. Ex-type strains are marked with *. The scale bar represents the expected number of nucleotide substitutions per site. The tree is rooted with *D. eres* (AR5193 and DLR12a).


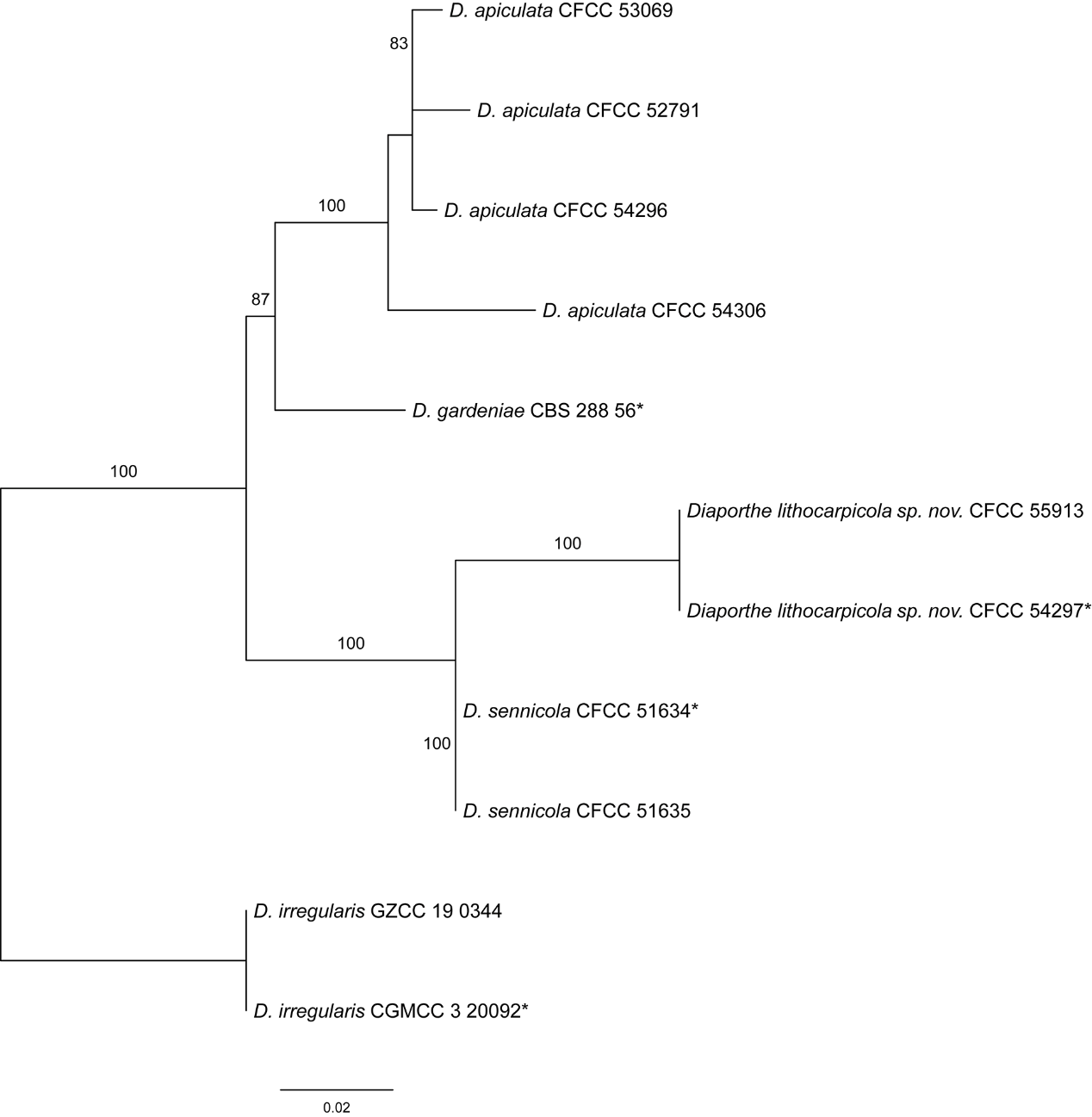


**Fig.** **S6** Phylogram of *Diaporthe gardeniae* species complex resulting from a maximum likelihood analysis based on the *cal* gene. Numbers above the branches indicate ML bootstrap values. Ex-type strains are marked with *. The scale bar represents the expected number of nucleotide substitutions per site. The tree is rooted with *D. irregularis* (CGMCC 3.20092 and GZCC 19-0344).


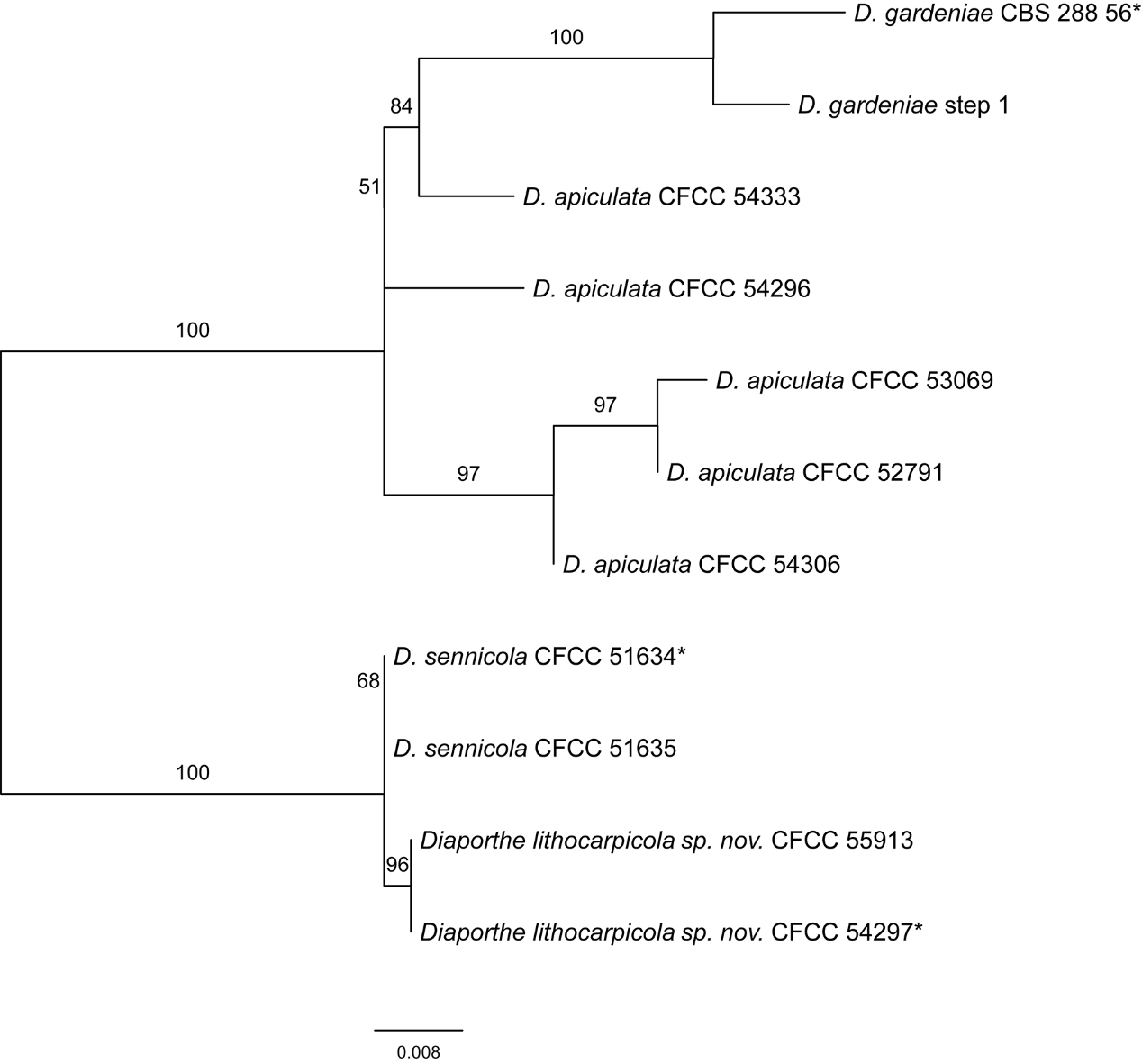


**Fig.** **S7** Phylogram of *Diaporthe gardeniae* species complex resulting from a maximum likelihood analysis based on the *his3* gene. Numbers above the branches indicate ML bootstrap values. Ex-type strains are marked with *. The scale bar represents the expected number of nucleotide substitutions per site. The tree is rooted with *D. irregularis* (CGMCC 3.20092 and GZCC 19-0344).


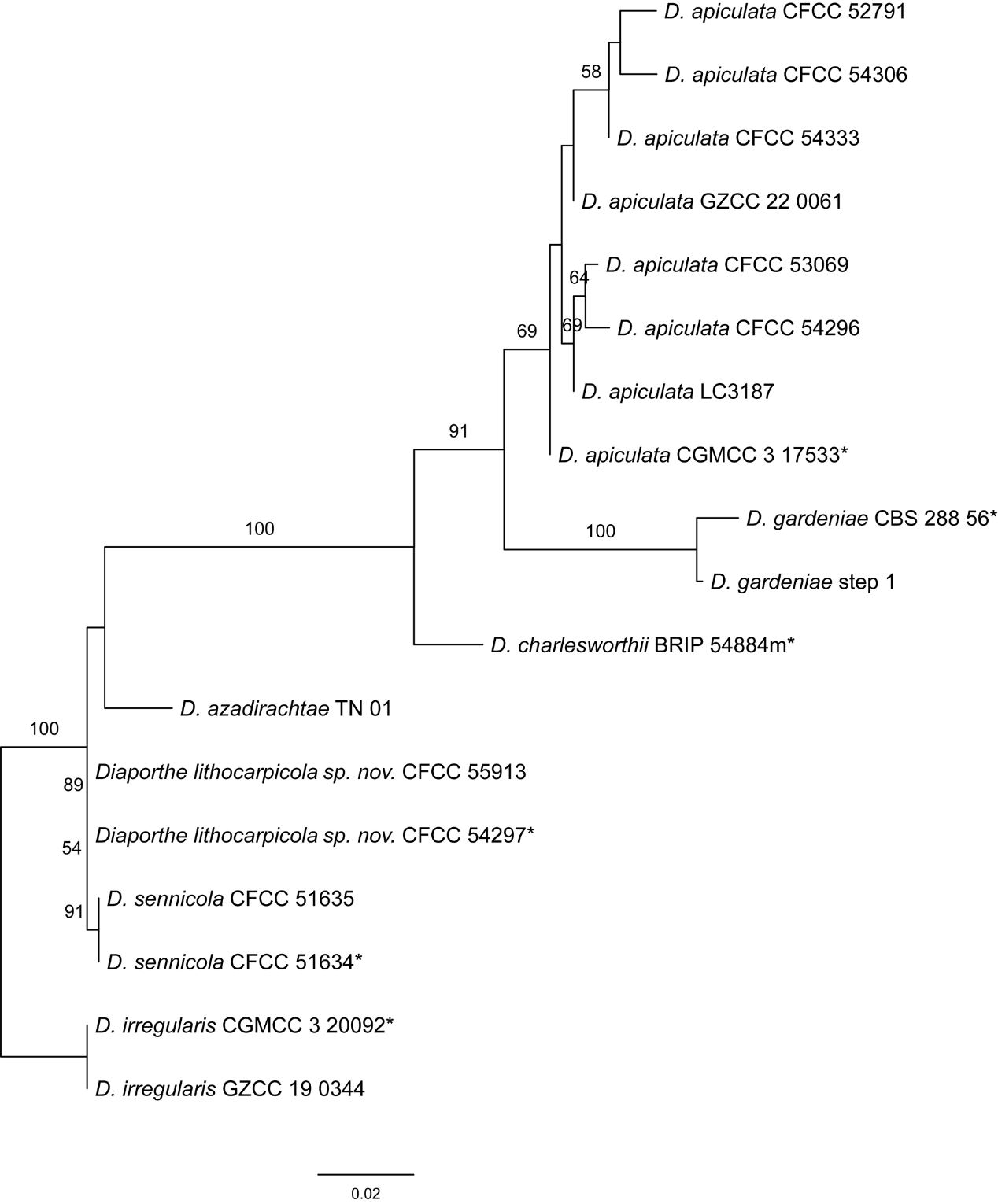


**Fig.** **S8** Phylogram of *Diaporthe gardeniae* species complex resulting from a maximum likelihood analysis based on the ITS sequence. Numbers above the branches indicate ML bootstrap values. Ex-type strains are marked with *. The scale bar represents the expected number of nucleotide substitutions per site. The tree is rooted with *D. irregularis* (CGMCC 3.20092 and GZCC 19-0344).


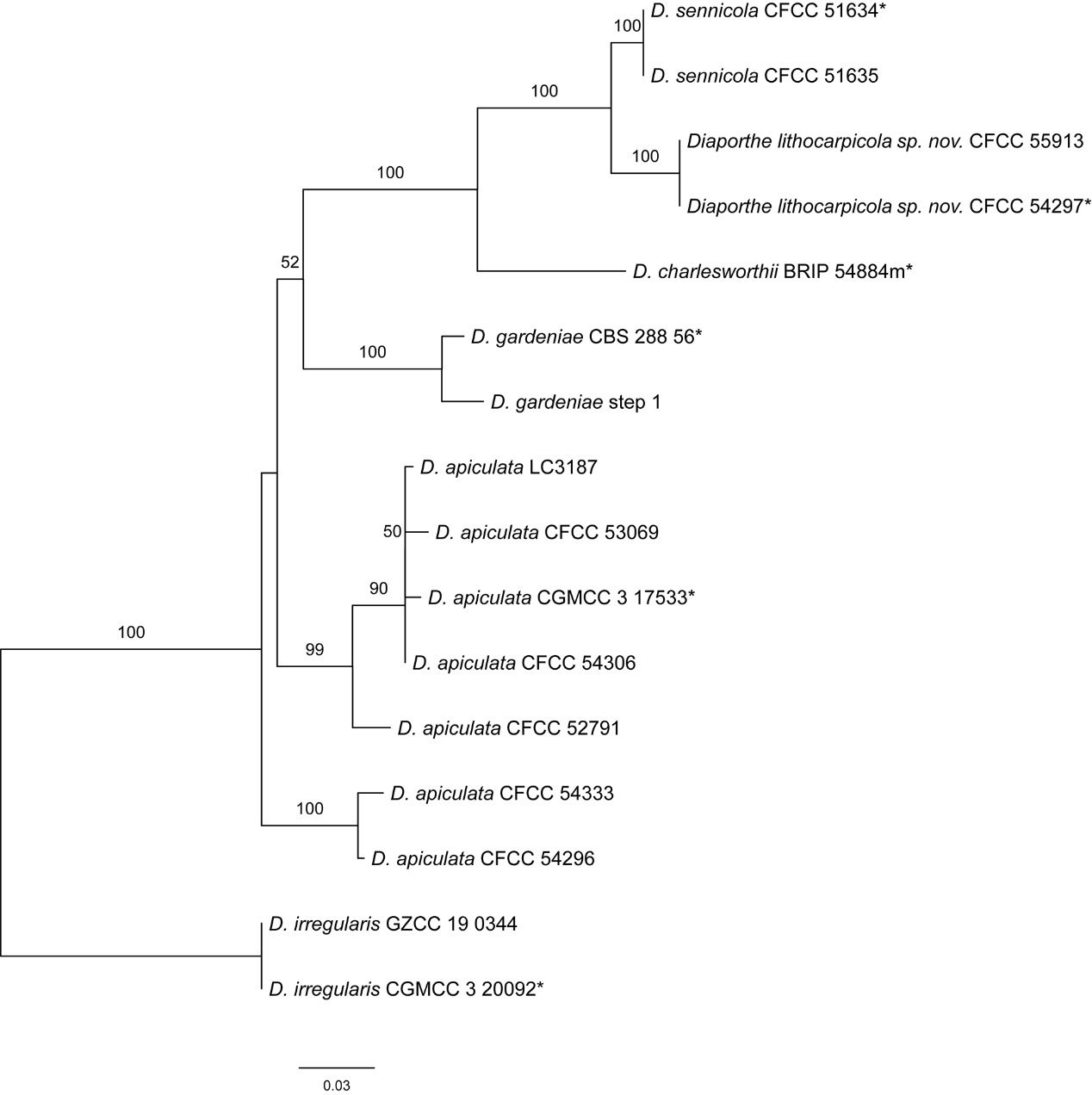


**Fig.** **S9** Phylogram of *Diaporthe gardeniae* species complex resulting from a maximum likelihood analysis based on the *tef1* gene. Numbers above the branches indicate ML bootstrap values. Ex-type strains are marked with *. The scale bar represents the expected number of nucleotide substitutions per site. The tree is rooted with *D. irregularis* (CGMCC 3.20092 and GZCC 19-0344).


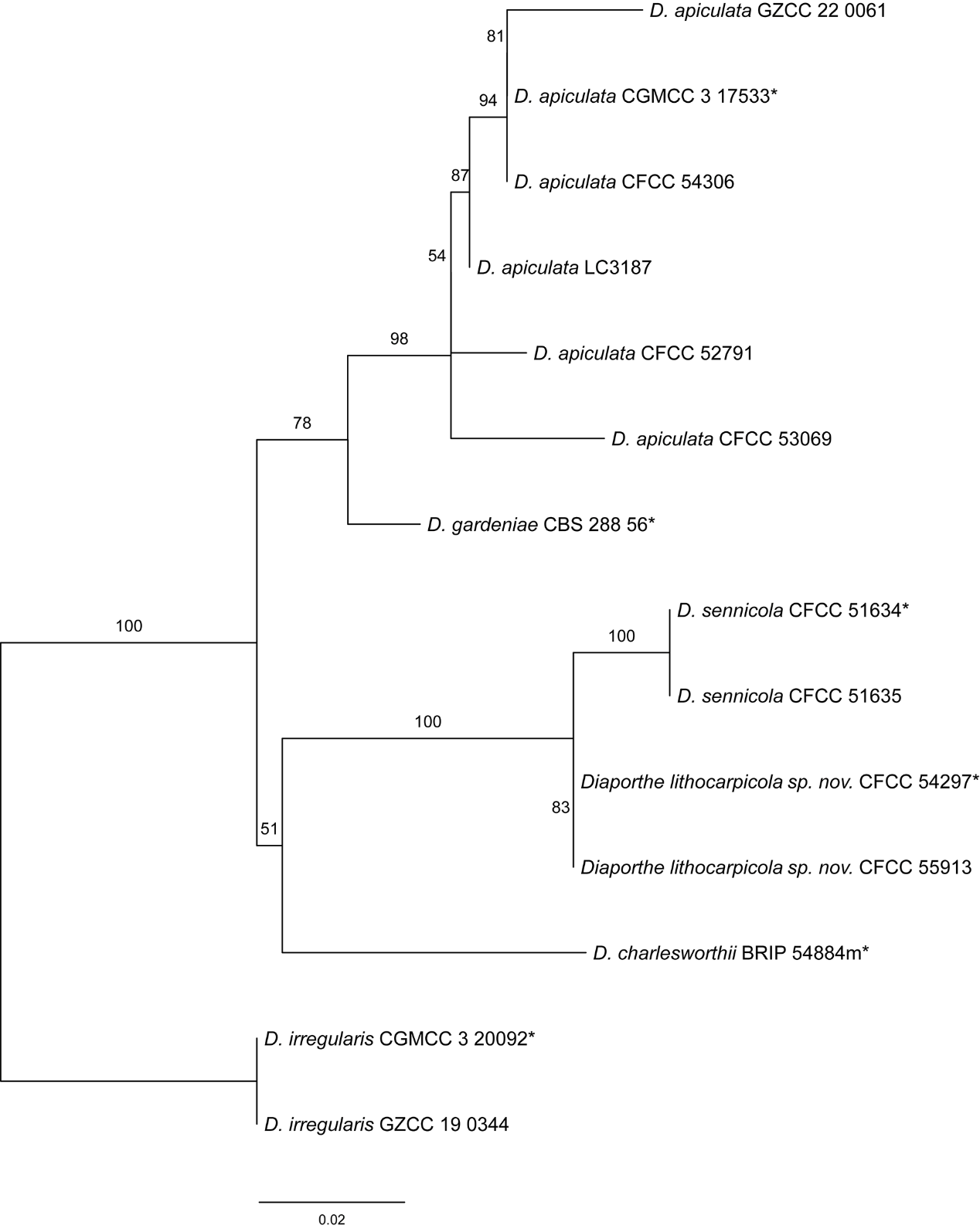


**Fig.** **S10** Phylogram of *Diaporthe gardeniae* species complex resulting from a maximum likelihood analysis based on the *tub2* gene. Numbers above the branches indicate ML bootstrap values. Ex-type strains are marked with *. The scale bar represents the expected number of nucleotide substitutions per site. The tree is rooted with *D. irregularis* (CGMCC 3.20092 and GZCC 19-0344).


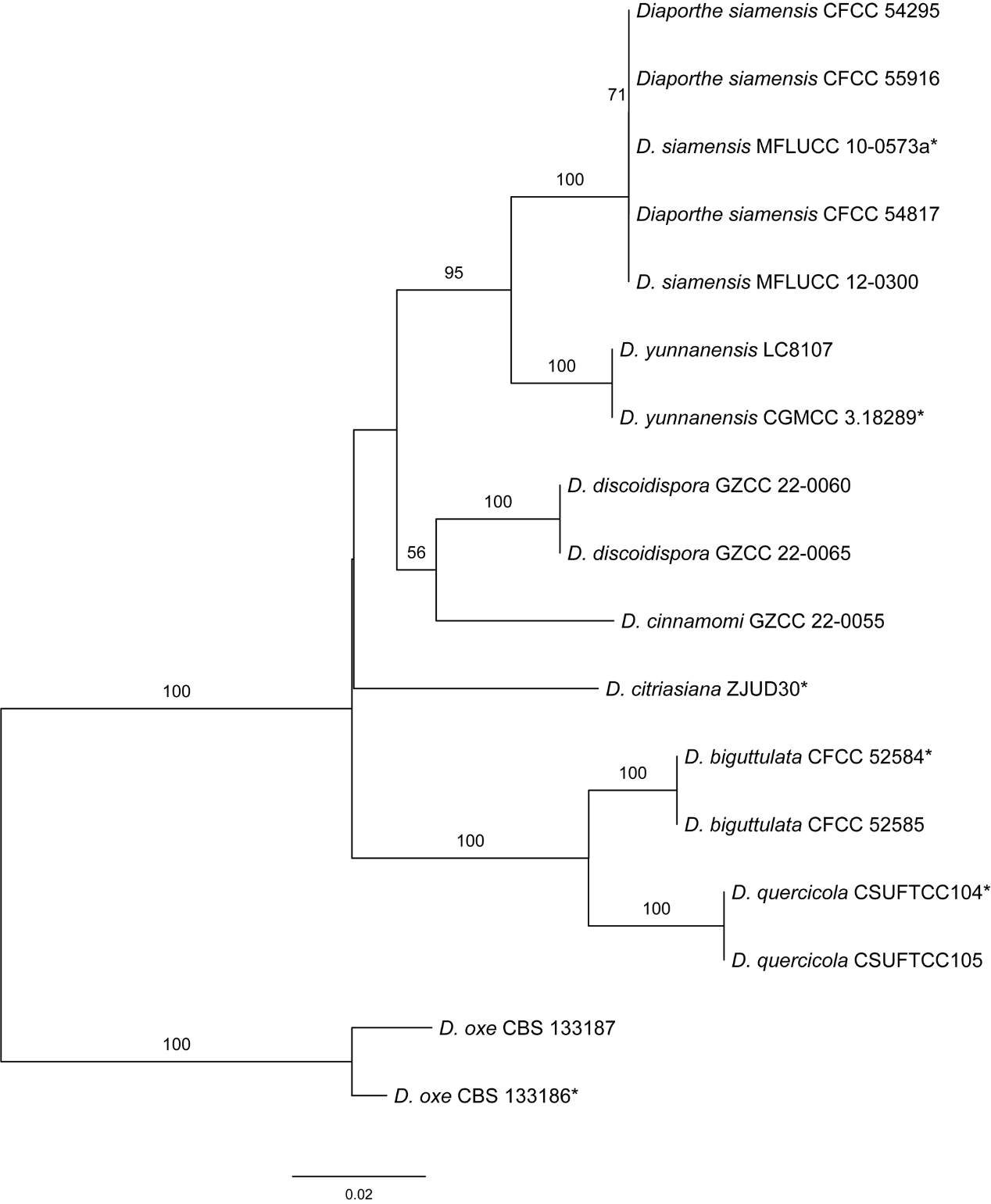


**Fig.** **S11** Phylogram of *Diaporthe siamensis* species complex resulting from a maximum likelihood analysis based on the *cal* gene. Numbers above the branches indicate ML bootstrap values. Ex-type strains are marked with *. The scale bar represents the expected number of nucleotide substitutions per site. The tree is rooted with *D. oxe* (CBS 133186 and CBS 133187).


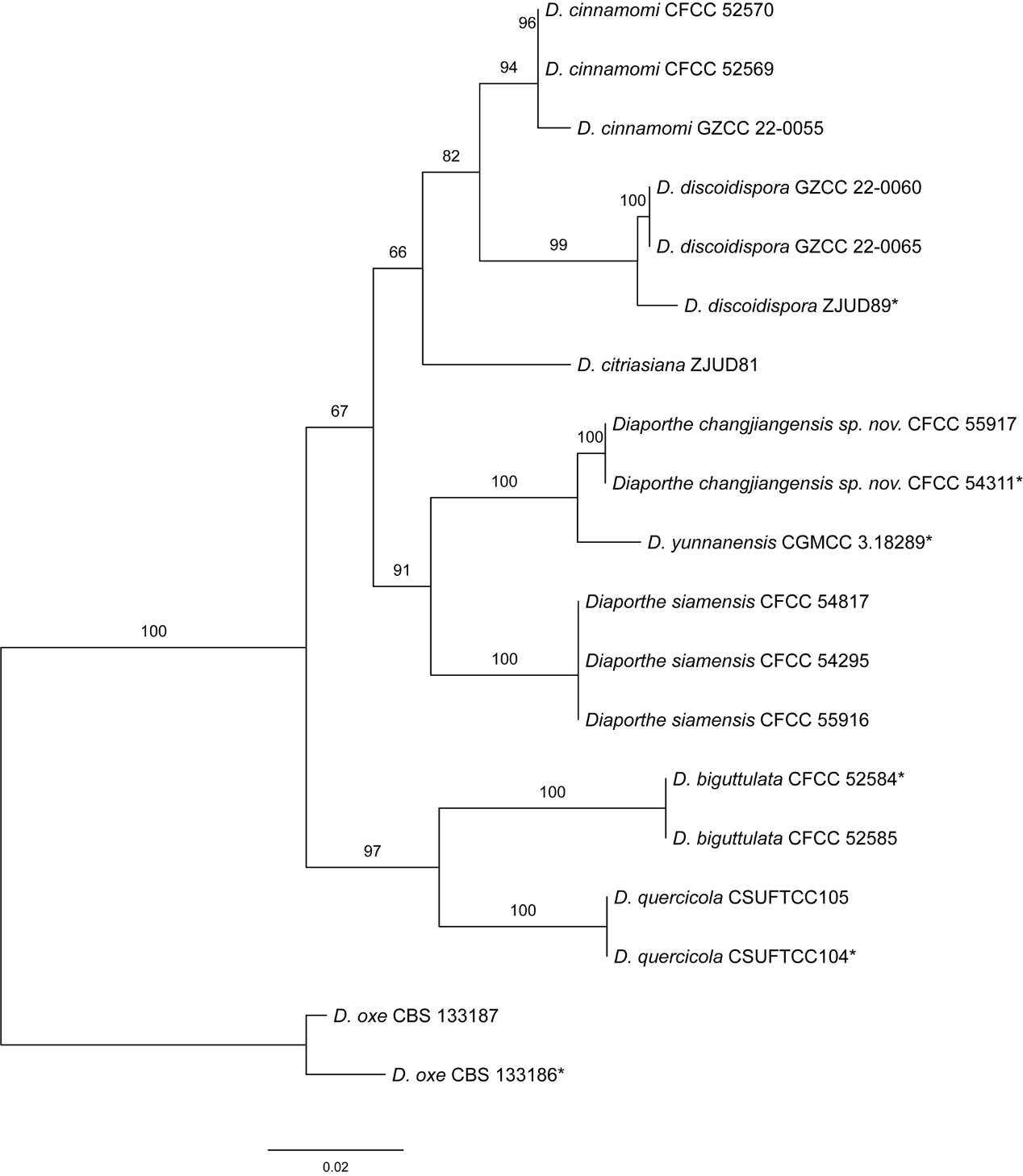


**Fig.** **S12** Phylogram of *Diaporthe siamensis* species complex resulting from a maximum likelihood analysis based on the *his3* gene. Numbers above the branches indicate ML bootstrap values. Ex-type strains are marked with *. The scale bar represents the expected number of nucleotide substitutions per site. The tree is rooted with *D. oxe* (CBS 133186 and CBS 133187).


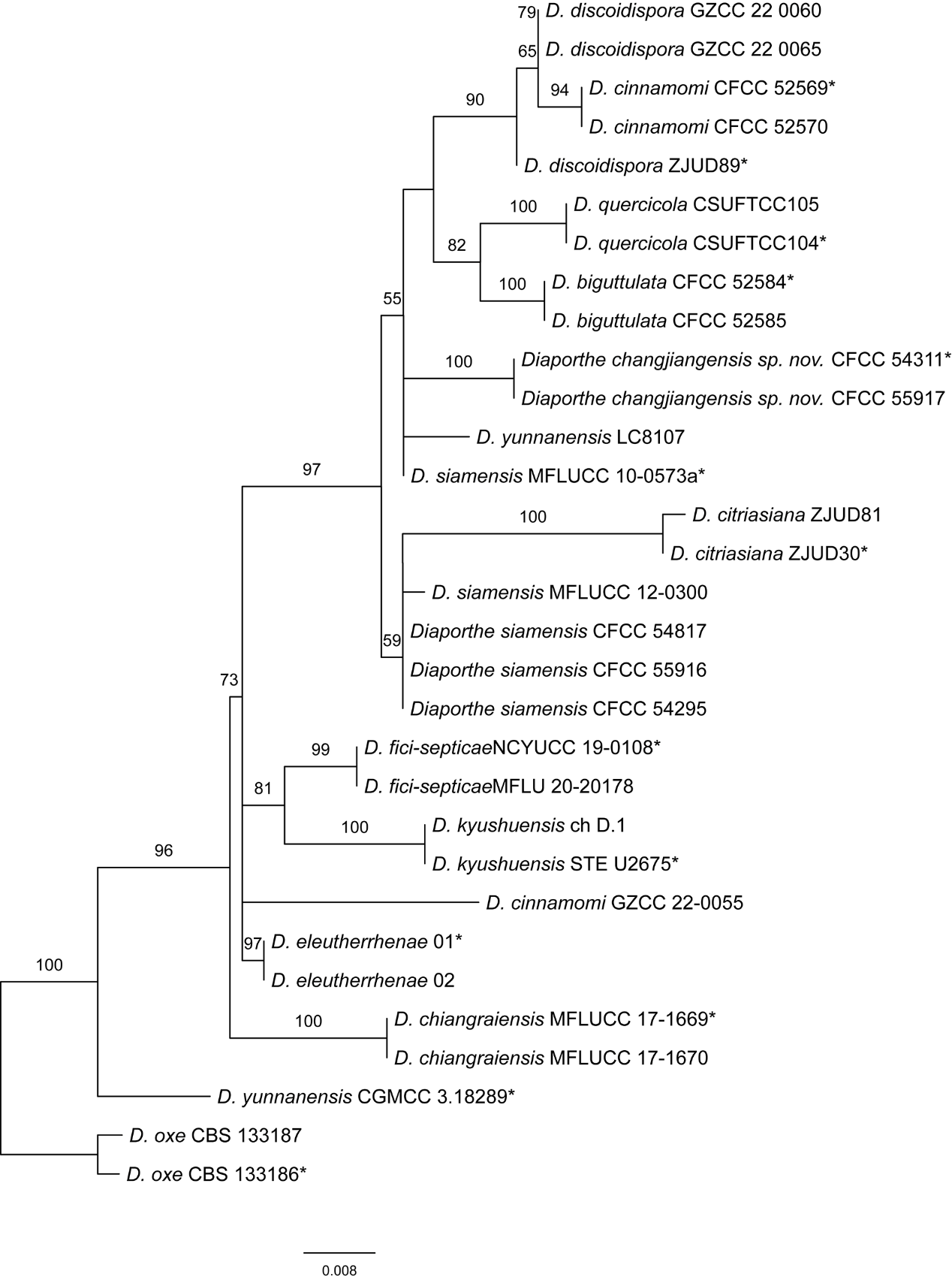


**Fig.** **S13** Phylogram of *Diaporthe siamensis* species complex resulting from a maximum likelihood analysis based on the ITS sequence. Numbers above the branches indicate ML bootstrap values. Ex-type strains are marked with *. The scale bar represents the expected number of nucleotide substitutions per site. The tree is rooted with *D. oxe* (CBS 133186 and CBS 133187).


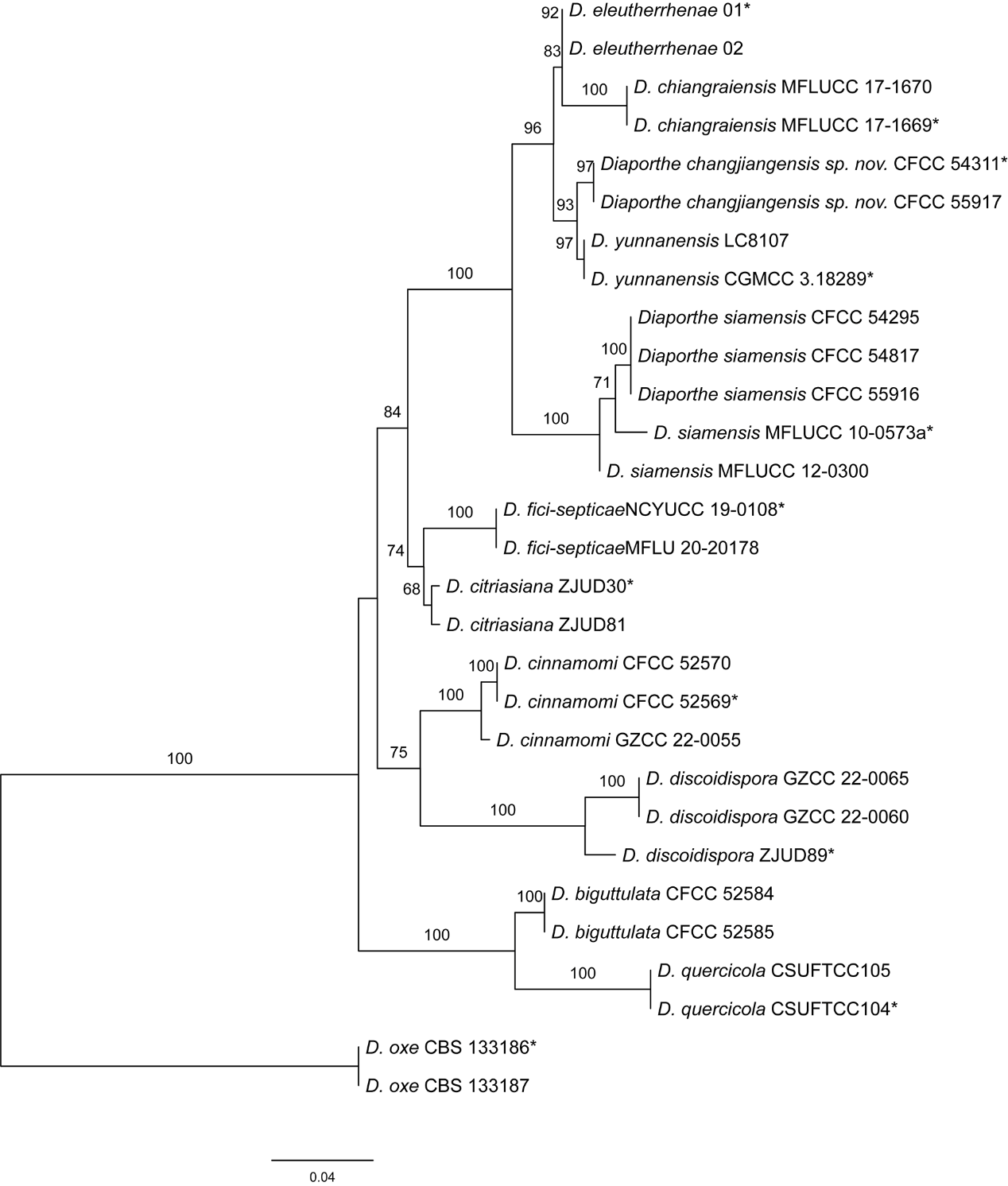


**Fig.** **S14** Phylogram of *Diaporthe siamensis* species complex resulting from a maximum likelihood analysis based on the *tef1* gene. Numbers above the branches indicate ML bootstrap values. Ex-type strains are marked with *. The scale bar represents the expected number of nucleotide substitutions per site. The tree is rooted with *D. oxe* (CBS 133186 and CBS 133187).


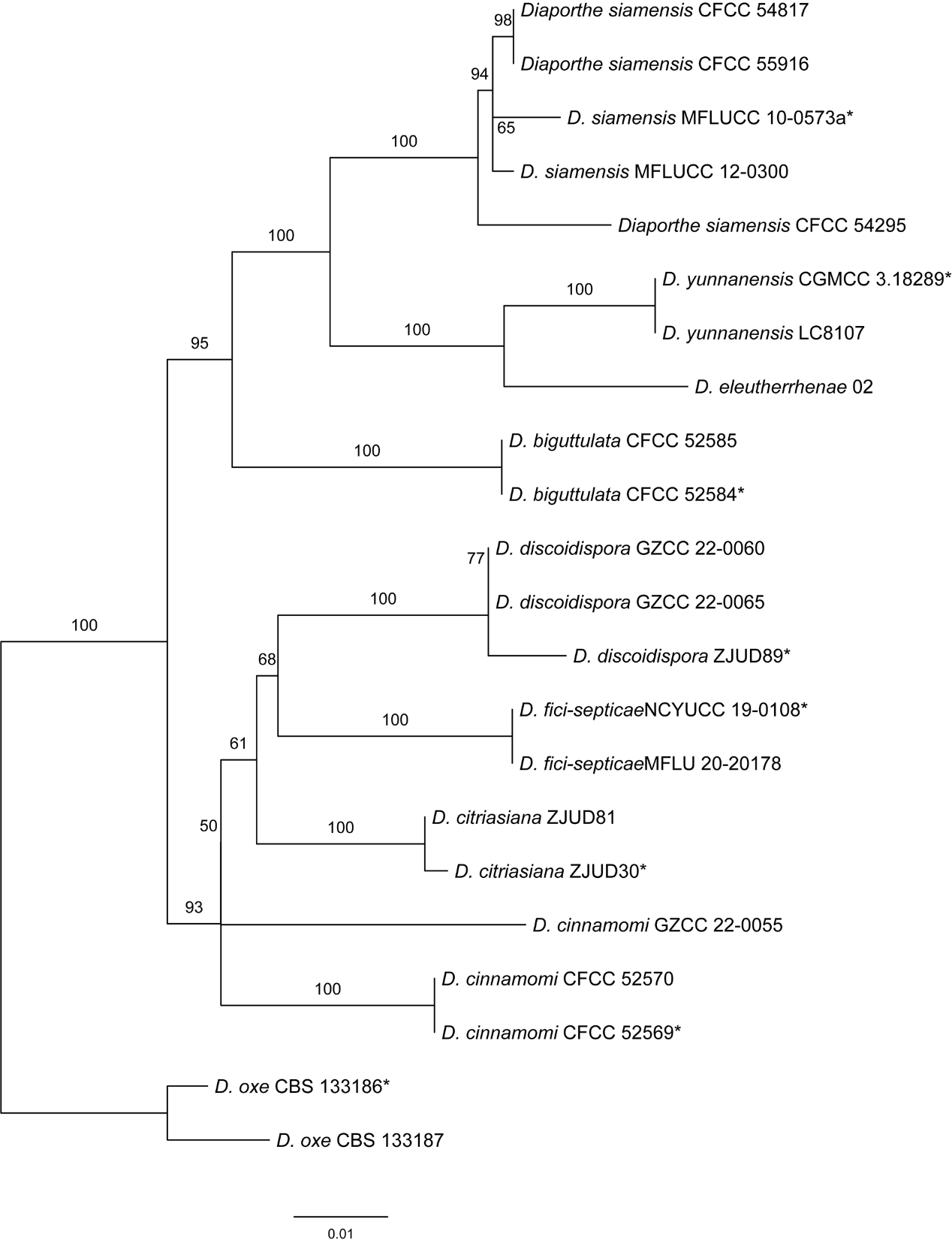


**Fig.** **S15** Phylogram of *Diaporthe siamensis* species complex resulting from a maximum likelihood analysis based on the *tub2* gene. Numbers above the branches indicate ML bootstrap values. Ex-type strains are marked with *. The scale bar represents the expected number of nucleotide substitutions per site. The tree is rooted with *D. oxe* (CBS 133186 and CBS 133187).


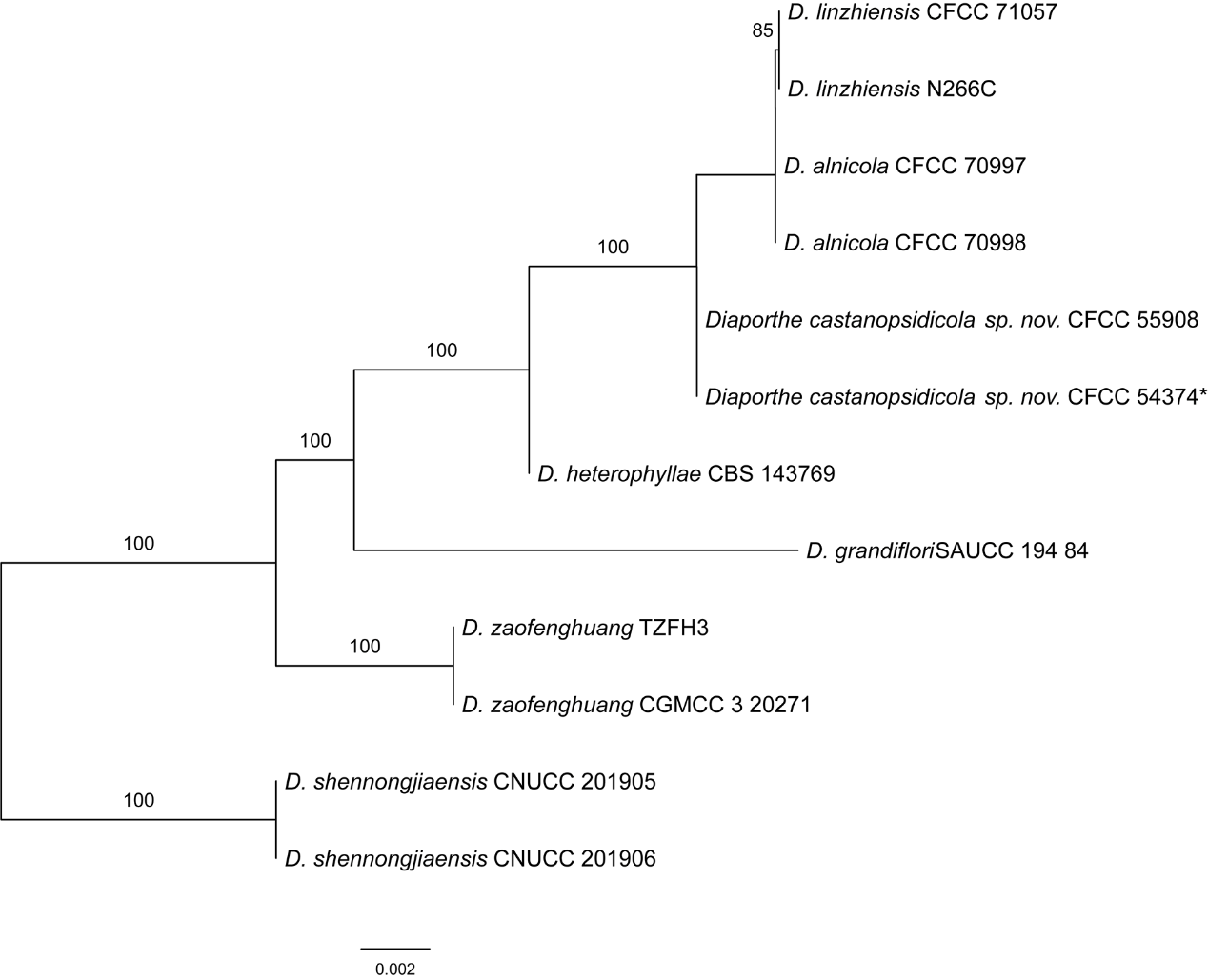


**Fig.** **S16** Phylogram of *Diaporthe virgiliae* species complex resulting from a maximum likelihood analysis based on the *cal* gene. Numbers above the branches indicate ML bootstrap values. Ex-type strains are marked with *. The scale bar represents the expected number of nucleotide substitutions per site. The tree is rooted with *D. shennongjiaensis* (CNUCC 201905 and CNUCC 201906).


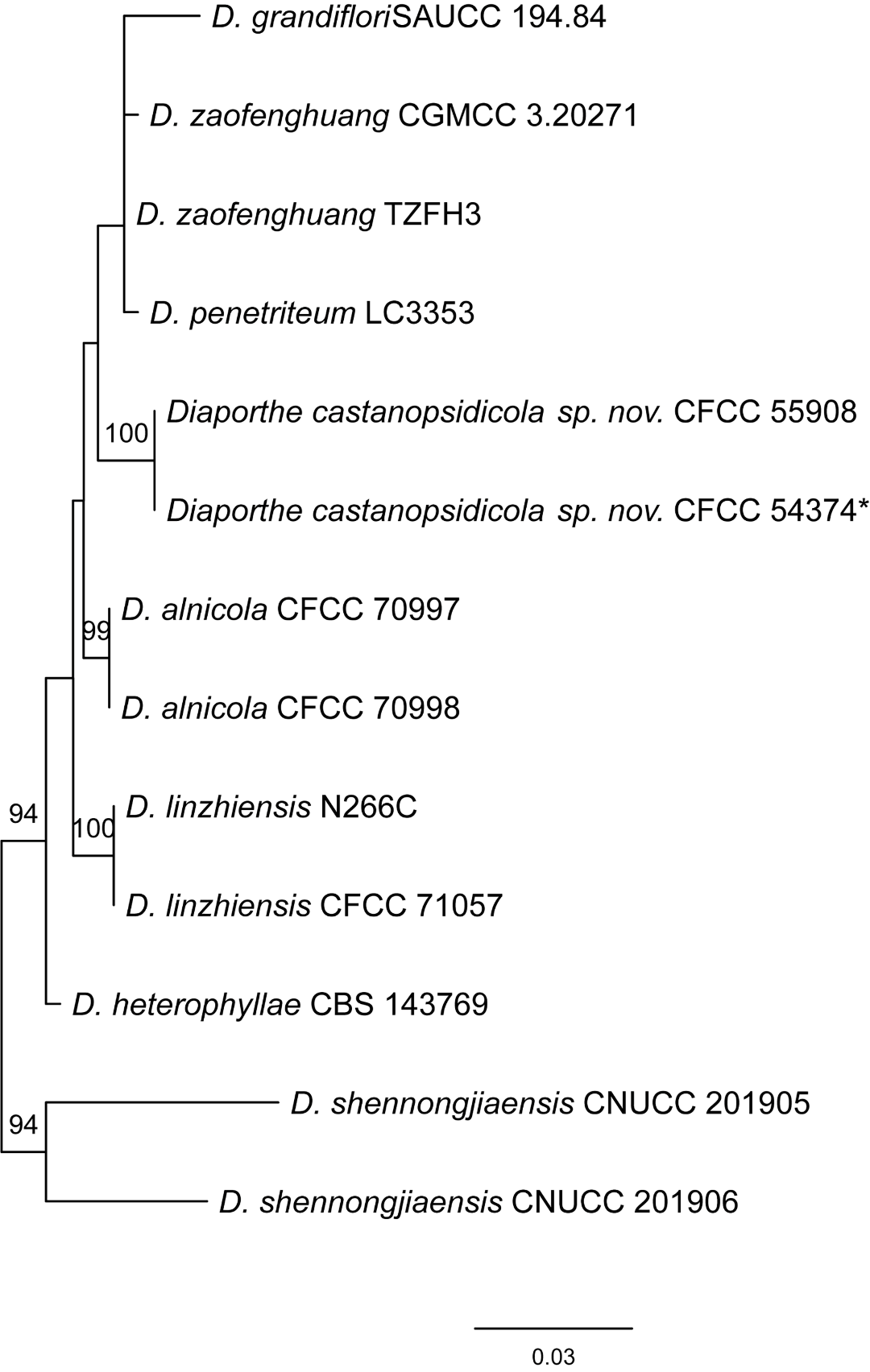


**Fig.** **S17** Phylogram of *Diaporthe virgiliae* species complex resulting from a maximum likelihood analysis based on the *his3* gene. Numbers above the branches indicate ML bootstrap values. Ex-type strains are marked with *. The scale bar represents the expected number of nucleotide substitutions per site. The tree is rooted with *D. shennongjiaensis* (CNUCC 201905 and CNUCC 201906).


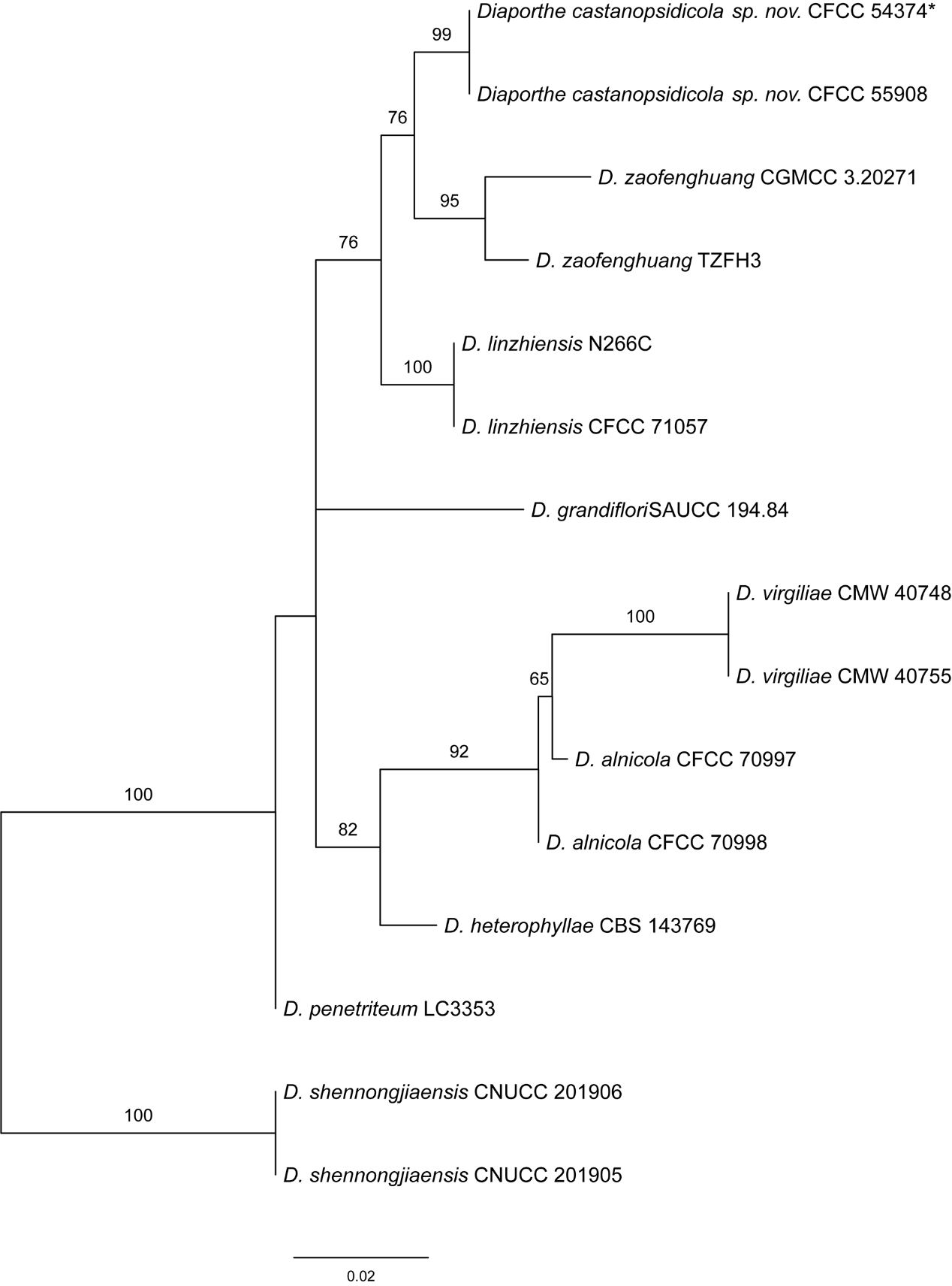


**Fig.** **S18** Phylogram of *Diaporthe virgiliae* species complex resulting from a maximum likelihood analysis based on the ITS gene. Numbers above the branches indicate ML bootstrap values. Ex-type strains are marked with *. The scale bar represents the expected number of nucleotide substitutions per site. The tree is rooted with *D. shennongjiaensis* (CNUCC 201905 and CNUCC 201906).


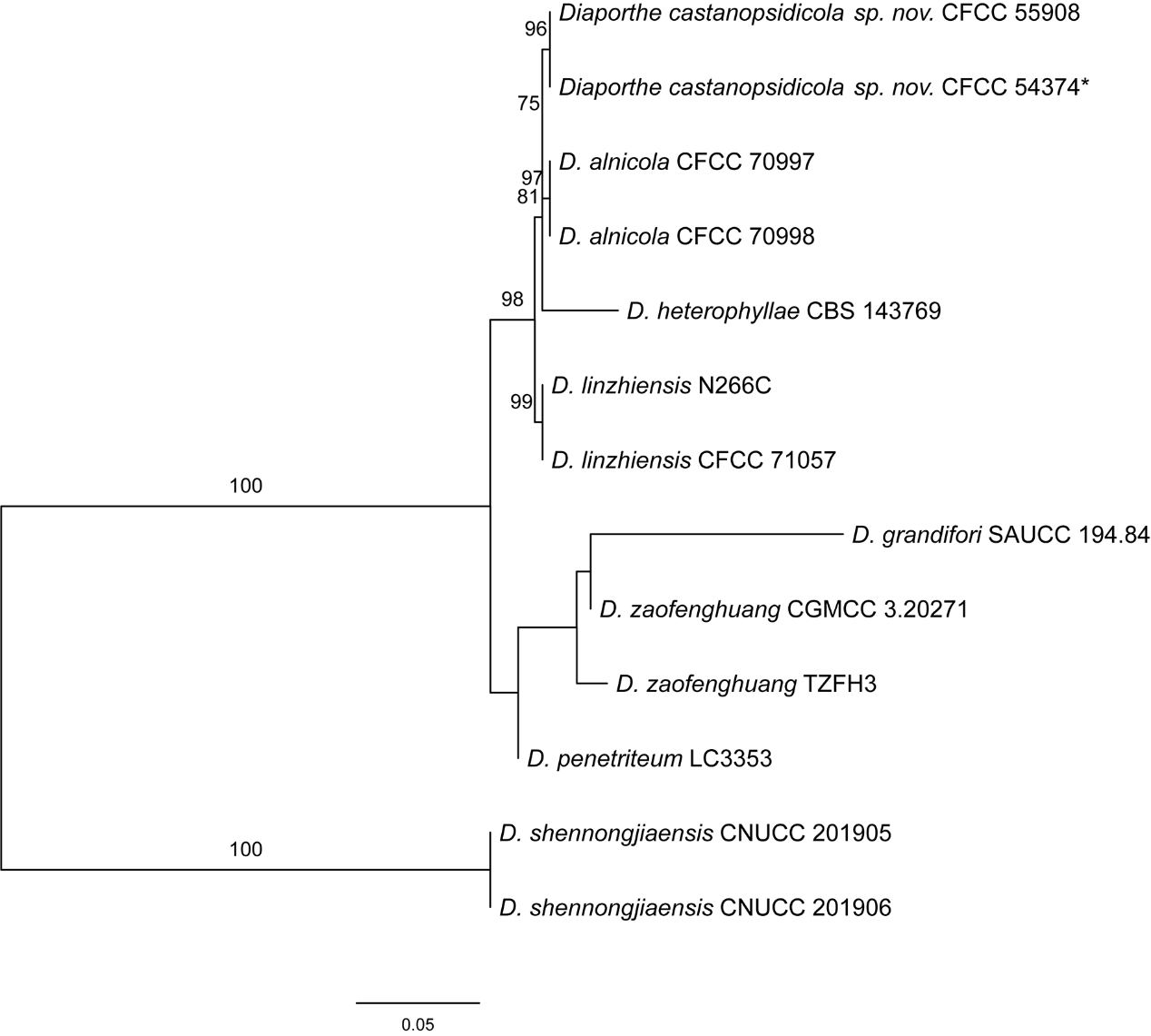


**Fig.** **S19** Phylogram of *Diaporthe virgiliae* species complex resulting from a maximum likelihood analysis based on the *tef1* gene. Numbers above the branches indicate ML bootstrap values. Ex-type strains are marked with *. The scale bar represents the expected number of nucleotide substitutions per site. The tree is rooted with *D. shennongjiaensis* (CNUCC 201905 and CNUCC 201906).


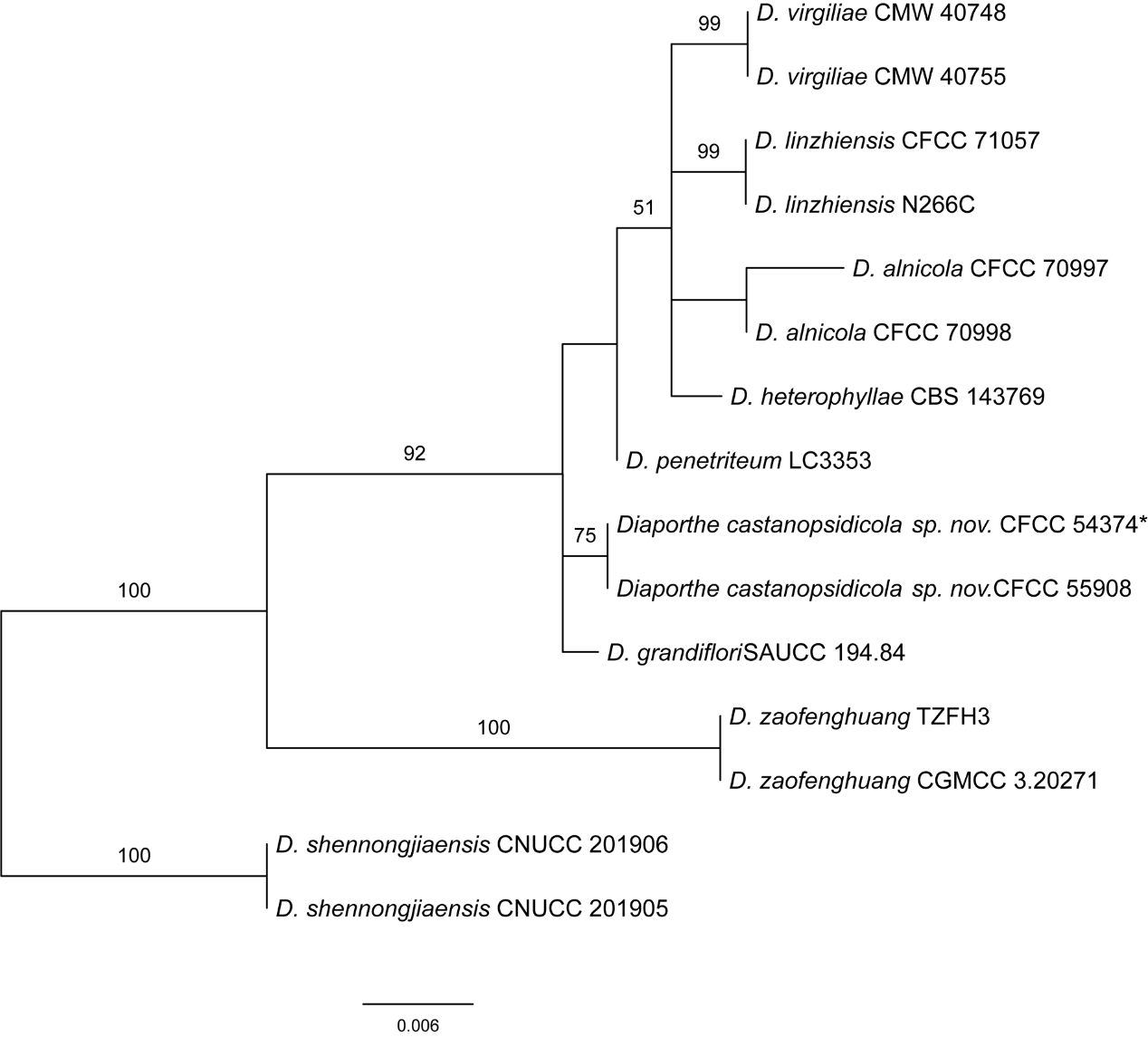


**Fig.** **S20** Phylogram of *Diaporthe virgiliae* species complex resulting from a maximum likelihood analysis based on the *tub2* gene. Numbers above the branches indicate ML bootstrap values. Ex-type strains are marked with *. The scale bar represents the expected number of nucleotide substitutions per site. The tree is rooted with *D. shennongjiaensis* (CNUCC 201905 and CNUCC 201906).
